# The effect of mobile phone messaging to support COVID-19 vaccination in Colombia: A randomized controlled clinical trial

**DOI:** 10.1371/journal.pgph.0006387

**Published:** 2026-06-11

**Authors:** Andres Ignacio Vecino-Ortiz, Deivis Nicolas Guzman-Tordecilla, Vidhi Maniar, Sandra Agudelo-Londoño, Nathaly Aya Pastrana, Oscar Franco Suárez, Mariana Rodríguez Patarroyo, Marino Mejia-Rocha, Mariangela Chavez Chamorro, Jose Luis Ortiz Hoyos, Antonio J. Trujillo, Joseph Ali, Dustin G. Gibson

**Affiliations:** 1 Department of Health, Behavior & Society. Johns Hopkins Bloomberg School of Public Health, Baltimore, Maryland, United States of America; 2 Instituto de Salud Pública Pontificia Universidad Javeriana, Bogotá, Colombia; 3 IMEK Centro de Investigación en Mercadeo & Desarrollo Santiago de Cali, Colombia, Colombia; 4 Inter-American Development Bank, Bogota, Colombia; PLOS: Public Library of Science, UNITED STATES OF AMERICA

## Abstract

During the COVID-19 pandemic, the speed required and the complexity of the information provided about vaccines highlighted the relevance of communicating evidence effectively during a sanitary emergency. We tested three interactive voice response (IVR) interventions, plus one control to increase COVID-19 vaccine uptake in an adult population in Colombia. We conducted a parallel-group randomized controlled trial with four arms in Colombia between September and December 2022. Participants (n = 2,409) were recruited via random-digit dialing and followed for 7 weeks. Eligible individuals were adults who were not fully vaccinated against COVID-19 at baseline. Participants were randomized to a control arm or to one of three IVR message interventions: factual, narrative, or mixed (factual + narrative). The outcome measured was vaccine uptake during the assessment period. The intervention was provided by the engageSPARK platform. We used logistic regression and Cox proportional hazards models to evaluate the effectiveness of the intervention. Total observed follow-up exceeded 5,775 person-contacts. Exposure to any intervention was associated with higher odds of vaccination among participants who were not fully vaccinated at baseline and correctly answered the comprehension test (odds ratio: 1.40; 95% CI: 1.05-1.87), but not among those with no prior vaccine doses. In Cox models, assignment to any intervention was associated with a significantly higher hazard of vaccination compared with control (hazard ratio [HR]: 1.50; 95% CI: 1.24-1.83). Stratified analyses showed a substantially higher vaccination rate among those with at least one vaccine at baseline (HR: 1.53; 95% CI: 1.25-1.88). All intervention arms showed similar effectiveness. This IVR-based intervention was effective in accelerating COVID-19 vaccination uptake among adults with at least one vaccine, highlighting the importance of prior acceptance to the vaccine. Message comprehension and intervention intensity determine intervention effectiveness. IVR-based interventions represent a scalable and potentially cost-effective tool for supporting vaccination in low- and middle-income countries.

## Introduction

The COVID-19 pandemic required governments to seek effective vaccines against the SARS-CoV-2 virus. COVID-19 vaccines proved to be successful in reducing deaths, hospitalizations, and economic loss [1,2]. However, the fact that new vaccines were available less than a year after the pandemic was declared, that some of them involved new mRNA technology, the conflicting information around them, and the very same fact that this was a sanitary emergency, led to heightened vaccine hesitancy all around the world [3–5].

With COVID-19 vaccines, there were clear issues with supply, but also with demand. Many individuals were hesitant to receive vaccines authorized under emergency use provisions, especially those based on a new technology, mRNA [3–5]. To increase COVID-19 vaccine uptake, some countries used different strategies, including broadcasting the benefits of getting the vaccine [6], sending personal reminders [7], offering incentives [8], restricting access to some services through mandates [9], or using financial sanctions [10].

In particular, by leveraging the widespread use of mobile phone technology, some countries used text messages to nudge individuals to get vaccinated [10]. Such a strategy has proven effective in standard vaccination settings [11–14]. Although previous studies have found significant improvements in immunization uptake through mobile phone reminders, the COVID-19 vaccine is different due to concerns about its novelty, safety, widespread mistrust of government, and the speed of its introduction, raising the question of whether standard nudging strategies would produce the same effect. We are only aware of a previous study in the United States (US) evaluating the effect of an intervention using text messages during the COVID-19 pandemic with a modest effect [15]. However, to our knowledge, there are no previous studies assessing the effect of an intervention using voice messaging in a lower and middle-income country (LMIC).

In 2015, Colombia had almost 1.2 mobile phones per inhabitant [16] and mobile technology penetration reached 80% in 2023. Mobile phone technology penetration in LMICs has facilitated communication in many areas, including finance. Therefore, high mobile phone technology coverage serves as an opportunity to contact the general public and provide information about vaccines and other health information during health emergencies that require social distancing [17]. Nonetheless, mobile phone technology also has limitations; for example, mobile phone technology is less likely to capture older and rural populations [16,18].

This study aimed to evaluate the effectiveness of three different mobile phone message interventions plus a control using interactive voice response (IVR) on vaccine uptake among Colombian adults through providing information about COVID-19 vaccine’s safety and efficacy.

## Methods

### Ethics statement

This study was approved by the Institutional Review Board at Johns Hopkins Bloomberg School of Public Health under protocol number 17868 and by the Ethics Committee of Pontificia Universidad Javeriana in Bogota, Colombia. The trial was registered at clinicaltrials.gov under protocol NCT05764174. The retrospective registration was due to the time related to institutional paperwork. Institutional IRB approval was finalized on September 20, 2021. The authors confirm that all ongoing and related trials for this intervention are registered.

This study was conducted, analyzed, and reported using the CONSORT guidelines, and the authors had no access to personally identifying data [19].

### Participants, consent, and recruitment

We conducted a parallel-group, superiority randomized controlled trial in Colombia between September 21^st^ and December 17^th^, 2022, to investigate the impact of different IVR message interventions on self-reported COVID-19 vaccination. The trial consisted of three treatment message arms and one control arm. All arms used IVR, that is, participants were called using prerecorded, interactive phone messages. Initial IVR surveys were developed, and cognitive testing was performed [20] using the IVR engageSPARK platform. These surveys used a syndromic surveillance instrument to recruit trial participants. The study detailing the methods for the syndromic surveillance instrument can be seen at Vecino-Ortiz et al [21]. In short, in the syndromic surveillance instrument, participant phone numbers were generated through random digit dialing (RDD) [22], which included a prefix ranging from 300 to 323 (which are the prefixes for all mobile phone numbers used in Colombia), followed by seven digits randomly selected. RDD is a technique used in research to yield a random sample of phone numbers when a previous database does not exist or cannot be accessed [23].

Informed consent was required for all trial participants. Potential participants were provided with pertinent information about the trial over the mobile phone through a pre-recorded audio message. After listening to this information, those interested in participating were invited to indicate their willingness to participate by pressing a designated digit on their mobile devices. This authorization was electronically recorded by the platform where all survey questions were recorded. This consent strategy has been implemented previously in similar studies, and has been studied carefully in the bioethics literature, including in the specific context of Colombia, where consent must also follow the current data privacy law [16,24–30].

All respondents to the surveillance instrument study who were 1) ≥18 years, 2) provided explicit consent according to previous research on consent strategies for mobile health surveys [24], and 3) agreed to participate in the surveillance study, were included. Eligible participants completed a 10-minute survey that collected information on sociodemographic status, respiratory symptoms, previous COVID-19 infection and vaccination status, preferences about COVID-19 vaccination, and information on COVID-19. Upon survey completion, respondents were given an airtime incentive of 4,000 Colombian pesos (around 1 USD at the time) [31] and, if eligible, were invited to participate in the IVR trial. The following additional inclusion criteria defined specific eligibility for the trial: 1) explicit willingness and consent to participate in the trial after having completed the surveillance instrument; 2) having not been fully vaccinated against COVID-19 for their age at the time of the trial (either those who did not get any vaccine, did not get the complete vaccination regimen including one booster if less than 50 years old or two boosters if older than 50 years old). Exclusion criteria was: withdrawing consent to participate in the trial at any time.

### Sampling and duration of the trial

Participants were randomly allocated to the four study arms using simple randomization, with a minimum target sample size estimated at N = 173 participants per arm completing the first survey. This allowed for the detection of a difference between groups of at least 15% with a double-tailed distribution at alpha = 0.05 and power = 0.8. The risk of attrition was unknown, given that this was the first time a trial like this had been carried out. Hence, we oversampled the treatment and control arms in two subsequent cohorts. In the first cohort, we aimed at recruiting 250 participants agreeing to participate in the trial on September 21, 2022 with a final sample size of 1,485 respondents agreeing to participate. A second cohort of participants was deployed on October 25, 2022 aimed at adding at least 150 more individuals per arm completing the first survey. The final sample increased by 924 respondents for a total of 2,409 respondents. The first cohort ended on November 26, 2022 and the second cohort ended on December 17, 2022.

### Intervention and control

Participants consenting to participate in the study were randomized to the control or one of the treatment arms and called twice a week during seven weeks (a total of 14 messages). Given the nature of the intervention, participants could not be blinded to which type of message they received. However, they were not informed of the existence or content of alternative message arms. Investigators were blinded to individual-level allocation during data collection and primary analyses, as all responses were de-identified, stored, and processed. Outcome assessment relied on standardized, automated IVR questions, minimizing interviewer or observer bias. In arm 1, participants received a factual message providing evidence-based information on the safety and effectiveness of the vaccine in a neutral tone described by a professional speaker. Arm 2 participants received a narrative message consisting of real-life testimonials delivered by a professional speaker about the vaccine’s safety and effectiveness, employing an emotional tone. Arm 3 participants received a mixed message, which collated factual messages in weeks 1, 3, 5, and 7, and narrative messages in week 2, 4, and 6. Factual and narrative messages are presented in S1 Table. Participants in the control arm did not receive any message intervention. All participants in the treatment arms were asked to respond to a single multiple-choice item assessing their understanding of the vaccination message, delivered immediately after it. All participants in both treatment and control arms were asked to briefly consent to the call, then inquired about COVID-19 vaccination in the last two weeks and were provided with a 4,000 COP (approximately 1 USD) incentive in mobile phone credit after completing every call (irrespective of answering the test correctly or not).

In summary, the treatment arms involved a total of seven different messages, repeated twice a week over a period of seven weeks. Each week, a new message was presented to participants. All participants were asked about their COVID-19 vaccination status, and a test was administered to check their understanding of the message content. Those in the control arms were only asked about their vaccination status (without a message and test) and then provided with the incentive.

The messages were co-designed using data from qualitative interviews among individuals recruited from the surveillance instrument where information on knowledge, perceptions, and vaccination experiences was collected. The messages were crafted in co-design sessions with the research team led by a scholar in social marketing. Details on the methodology of the co-design of the messages are published by Aya Pastrana et al [32].

### Outcome measures

Our primary outcome variable was receiving a COVID-19 vaccination following seven weeks of intervention messaging. Weekly, participants were asked if they had received a COVID-19 vaccination. Participants who did not complete the vaccination question in a given call were excluded from that specific analysis but remained in the dataset for subsequent assessments if they responded. No data imputation was performed.

### Statistical analysis

First, we conducted descriptive analyses to examine the distribution of sociodemographic characteristics across the different arms, ensuring proper randomization (Table 1). Second, we assessed the change in vaccination status according to the number of follow-up contacts or messages received during the study. Recognizing that the effects of interventions may vary based on the initial vaccination status, we segmented our models according to the vaccination status at baseline. This refers to the vaccination status at the beginning of the follow-up (first contact). The categories for the vaccination status were as follows:

- *Not fully vaccinated*: Individuals who were not fully vaccinated, were eligible for a second dose or booster but had not received it.
- *Had not received any dose*: Individuals who had not received any COVID-19 vaccine.

**Table 1:** Characteristics of participants in the control and treatment arms.

Variables	Cohort 1 (n = 1,485) % (n)	Cohort 2 (n = 924) % (n)	Factual (n = 805) %(n)	Narrative (n = 512) %(n)	Mixed (n = 511) %(n)	Control (n = 581) %(n)	Interventions (n = 1,828) %(n)	p-value
**Sex**								
Female	57.40% (845)	57.19% (525)	59.32% (471)	54.12% (276)	56.50% (287)	58.13% (336)	57.06% (1,034)	0.65
Male	42.60% (627)	42.81% (393)	40.68% (323)	45.88% (234)	43.5% (221)	41.87% (242)	42.94% (778)	0.65
**Age years** (mean)	37.28 (SD = 14.93)	37.92 (SD = 14.9)	37.40 (SD = 14.65)	38.81 (SD = 15.52)	37.58 (SD = 15.17)	36.54 (SD = 14.47)	37.84 (SD = 15.05)	0.06
**Age groups**								
18-29	37.64% (559)	33.66% (311)	36.65% (295)	32.81% (168)	35.62% (182)	38.73% (225)	35.28% (645)	0.13
30-44	32.86% (488)	34.85% (322)	32.67% (263)	31.84% (163)	34.25% (175)	35.97% (209)	32.88% (601)	0.16
45-59	20.47% (304)	21.75% (201)	22.11% (178)	23.24% (119)	20.94% (107)	17.38% (101)	22.1% (404)	0.01
60+	9.02% (134)	9.74% (90)	8.57% (69)	12.11% (62)	9.2% (47)	7.92% (46)	9.74% (178)	0.18
**Geographic area**								
Urban	74.68% (1,109)	71.43% (660)	73.66% (593)	69.34% (355)	72.60% (371)	77.45% (450)	72.16% (1,319)	0.01
Rural	25.32% (376)	28.57% (264)	26.34% (212)	30.66% (157)	27.40% (140)	22.55% (131)	27.84% (509)	0.01
**Education level**								
None/Primary	16.77% (249)	20.45% (189)	18.39% (148)	19.34% (99)	20.16% (103)	15.15% (88)	19.15% (350)	0.02
Secondary	65.52% (973)	66.56% (615)	66.83% (538)	67.58% (346)	67.32% (344)	61.96% (360)	67.18% (1,228)	0.02
College or more	17.71% (263)	12.99% (120)	14.78% (119)	13.09% (67)	12.52% (64)	22.89% (133)	13.68% (250)	<0.01

Note: *Interventions* refer to the intervention group for factual, narrative, and mixed. P-values were estimated between the *control* and *interventions* groups using hypothesis testing for each of the categories or for the continuous variable.

To explore the effect of the interventions on the change of vaccination status, we used logistic regression models. We first aimed at identifying the difference in vaccination rates between the control group and the treatment groups (those assigned to any treatment arm). Models 1 and 2 estimated the unadjusted association between intervention assignment and vaccination. Models 3 and 4 added an indicator for correctly answering the comprehension test. Next models adjusted for the number of messages (for treatment arms) or follow-up contacts (for the control arm) received (models 5 and 6). The number of messages is a continuous variable representing the number of messages received by participants in the intervention groups and asked about their vaccination status, or the number of times participants in the control group were asked about their vaccination status. Finally, we further adjusted the previous model to account for sociodemographic variables, including age (18–29, 30–44, 45–59, and 60+), sex, education level, geographic area of residence, and for whether the respondent was part of the first or second cohort to receive the message (models 7 and 8). Each of the three aforementioned models were performed separately for each vaccination status at baseline category (models 1,3,5,7 for those who “Had not received any dose”, and models 2, 4, 6, and 8 for those “Not fully vaccinated”. In total, we conducted eight logistic regressions (Table 1).

A similar analytical strategy was employed to explore the difference in vaccination rates for each specific arm compared to the control group (Factual vs. Control; Mixed vs. Control; and Narrative vs. Control). The models were stratified by vaccination status at baseline and adjusted using the same analytical strategy previously presented, starting with the message test and progressing to a full model that controlled for the test, number of messages, sociodemographic variables, and cohort. For each arm, we conducted six models, resulting in a total of 18 logistic regression models.

We conducted per-protocol analyses on the number of audio messages received and a sensitivity analysis by restricting our sample only to participants who successfully completed the knowledge test. We also assessed the probability of vaccination, controlling for answering the test correctly, the number of messages received, and sociodemographic conditions. Finally, we assessed attrition in the trial. Intervention fidelity was monitored through the IVR platform, which automatically recorded call attempts, successful message delivery, message completion, and responses to the comprehension test. The number of messages successfully delivered and completed by each participant was incorporated into per-protocol and dose–response analyses. Missing data were handled using a complete-case approach in the analysis. For each follow-up contact, participants who did not respond to the vaccination status question were excluded from that analysis but remained eligible for subsequent analyses if they completed subsequent follow-up calls. No imputation of missing outcomes or covariates was performed.

This approach was chosen because missingness arose primarily from intermittent nonresponse and attrition inherent to IVR-based longitudinal follow-up rather than item-level refusal, and because the timing of vaccination events could still be accurately captured for participants who re-entered follow-up.

Primary analyses were conducted on a per-protocol basis, reflecting exposure to the intervention (i.e., the number of messages received). Sensitivity analyses were restricted to participants who completed the comprehension test. An intention-to-treat analysis was not feasible because exposure intensity (number of calls received) varied due to differential follow-up and call completion.

Model discrimination and calibration were assessed for the primary adjusted logistic regression model using the area under the ROC curve (AUC) and the Hosmer–Lemeshow test [33,34].

### Survival analysis

After the logistic regression analyses, we conducted a survival analysis to examine differences in the timing of COVID-19 vaccination across study arms. While logistic regression models estimate the overall probability of vaccination during follow-up, survival analysis explicitly incorporates time under observation, accounts for differential follow-up, and exploits information on when vaccination occurred rather than only whether it occurred.

Time to vaccination was defined as the number of follow-up contacts (twice a week during seven weeks) from the first outreach contact to the first report of COVID-19 vaccination. Because the exact calendar date of vaccination was not observed, follow-up contacts were used as a discrete proxy for time. Participants who did not report vaccination during the study period were censored at their last completed follow-up contact. Participants lost to follow-up were censored at their final observed contact.

Kaplan–Meier failure curves were used to describe the cumulative incidence of vaccination over time by study arm. Next, we estimated Cox proportional hazards models to quantify differences in the hazard of vaccination between intervention arms and the control group. Models were estimated for the full sample and stratified by vaccination status at baseline (either had not received any dose and not fully vaccinated). To further examine progression along the vaccination pathway, we conducted two additional conditional survival analyses. Among individuals who had received exactly one vaccine dose at baseline, we estimated a Cox model for second-dose uptake, where the event was defined by the completion of the primary vaccination series. Among individuals who had completed the primary vaccination series at baseline, we estimated a Cox model for booster uptake, where the event was receipt of the next eligible booster dose (first or second booster, depending on eligibility). In each model, the risk set was restricted to individuals eligible for the corresponding vaccination step at baseline. Hazard ratios (HRs) and 95% confidence intervals were reported. All models accounted for within-participant correlation by clustering standard errors at the individual level.

Fully adjusted models controlled for age group, sex, education level, geographic area of residence and cohort. The proportional hazards assumption was assessed using Schoenfeld residuals and was not violated in any model (global tests *p* > 0.60).

Survival analysis results were interpreted as complementary to the logistic regression findings, providing evidence on both the likelihood and the time to vaccination uptake in response to the interventions.

Study protocol is available in S1 Text. CONSORT checklist is available in S1 Checklist [19]. Inclusivity in Global Research Questionnaire is in S2 Checklist.

## Results

### Descriptive analysis

**Fig 1:**
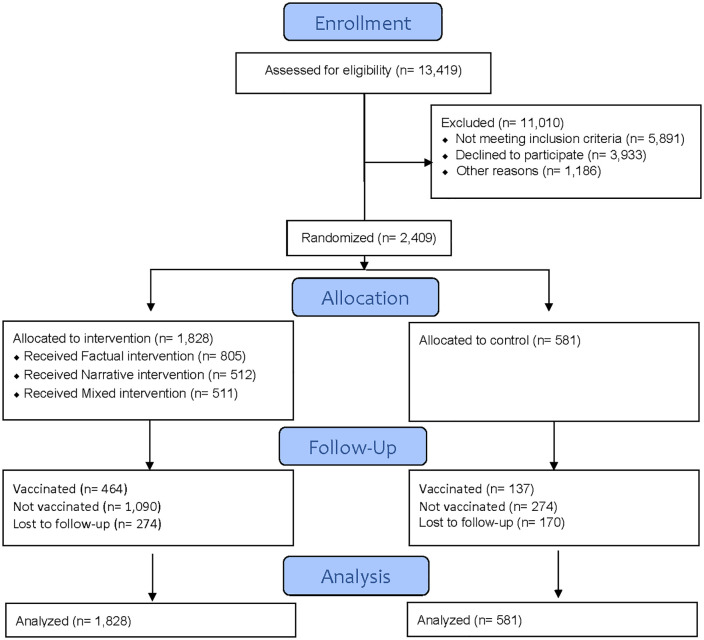
CONSORT Flow Diagram. Flow-chart showing inclusion, randomization and participation in the study. Note: In Fig 1, “Other reasons” include partial interviews, survey break-offs, technical call failures and incomplete baseline surveys that prevented confirmation of eligibility to the trial.

**Fig 2:**
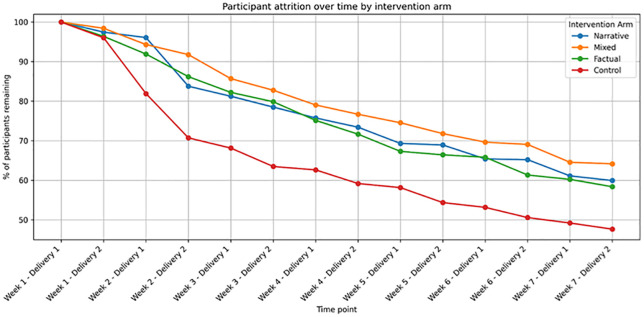
Participant attrition over time by intervention arm.

A total of 2,409 participants were included in the analysis (See Fig 1 for consort RCT flow). The sample consisted of 57% female participants, with an average age of 37.46 (SD = 14.66; range: 18–98). Seventy-three percent (73%) of participants resided in urban areas, and more than 82% had a high school degree or higher education. Overall, we found that characteristics of participants in the intervention and control groups were balanced (Table 1) except for education, as college graduates were more likely to be in the control arm. Attrition can be seen in Fig 2 and was more important in the control group (52%) compared to the treatment arms (36%–42%).

Participants who withdrew from the study were slightly younger (average age 37.18 vs. 37.46 years), had a similar proportion of females (57%), a higher proportion from urban areas (75% vs. 73%), and a comparable percentage with at least a high school degree or higher education (82%).

**Fig 3:**
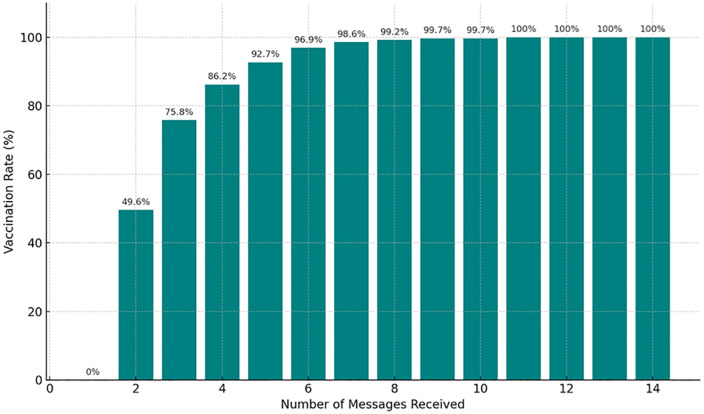
Vaccination rate by message delivery (1-14) among those in the treatment arms who ultimately vaccinated. Note: This figure displays the cumulative vaccination rate among participants who report having been vaccinated across the number of follow-up messages received in the intervention group.

**Fig 4:**
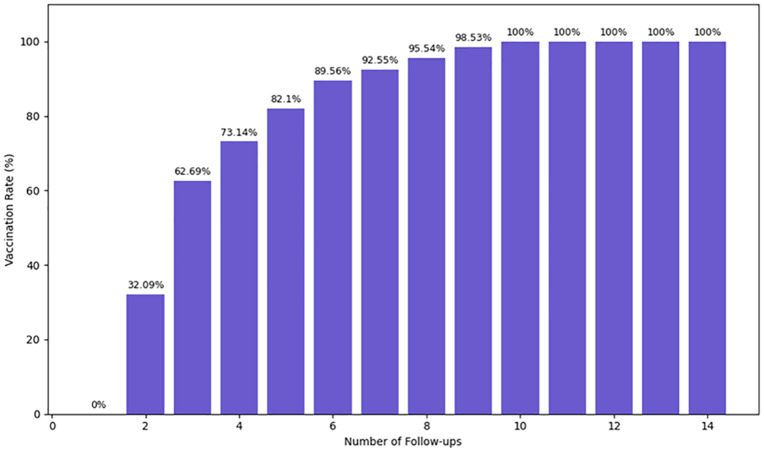
Vaccination rate by message delivery (1-14) among those in the control arm who ultimately vaccinated. Note: This figure displays the cumulative vaccination rate among participants who report having been vaccinated across the number of follow-up messages received in the control group.

Vaccination rates increased progressively with the number of follow-up messages received in both the intervention and control groups. However, the intervention group showed a faster uptake, reaching a 86.2% vaccination rate after just four messages. In contrast, the control group achieved a vaccination rate of 62.7% after three messages. In Figs 3 and 4, we present the rate at which participants from both the treatment and control groups were vaccinated. 96.9% of individuals who got vaccinated in the treatment groups reported receiving vaccines by message 6, implying that a 3-week series of messages might be sufficient to reach herd immunity among those who are indeed sensitive to the intervention.

Given the low-risk nature of the intervention, adverse event monitoring was conducted via participant self-report during follow-up calls. No adverse events related to participation in the study or exposure to IVR messages were reported.

### Effect of treatment group on change of vaccination status

Without controlling for the understanding of the content of message or the actual number of messages received, the interventions had no measurable impact on vaccination compared to the control. However, we found that being in either the factual, narrative or mixed arm increased the vaccination OR by 1.40 (95% CI: 1.05 to 1.87), once adjusted for the message test and the number of messages/follow-up contacts received. Although the effect slightly decreased (OR: 1.36; 95% CI: 1.01 to 1.82) after adjusting for sociodemographic variables, the significance remained (p < 0.05). However, these results pertained only to individuals whose baseline vaccination status was “Not fully vaccinated.” No statistically significant effect was identified for those whose baseline was “Had not received any dose” (Table 2).

**Table 2:** Effect of the Intervention on new COVID-19 vaccination.

	Model 1	Model 2	Model 3	Model 4	Model 5	Model 6	Model 7	Model 8
**Variables**	**Odds Ratio** **(95% CI)**	**Odds Ratio** **(95% CI)**	**Odds Ratio** **(95% CI)**	**Odds Ratio** **(95% CI)**	**Odds Ratio** **(95% CI)**	**Odds Ratio** **(95% CI)**	**Odds Ratio** **(95% CI)**	**Odds Ratio** **(95% CI)**
**Vaccination status at baseline**	**Had not received any dose**	**Not fully vaccinated (at least one dose)**	**Had not received any dose**	**Not fully vaccinated (at least one dose)**	**Had not received any dose**	**Not fully vaccinated (at least one dose)**	**Had not received any dose**	**Not fully vaccinated (at least one dose)**
**Intervention *(reference group: control)***	0.36** (0.15 to 0.83)	0.86 (0.68 to 1.09)	0.47 (0.19 to 1.15)	1.11 (0.85 to 1.44)	0.49 (0.19 to 1.28)	1.40** (1.05 to 1.87)	0.50 (0.18 to 1.34)	1.36** (1.01 to 1.82)
**Test**			**Yes**	**Yes**	**Yes**	**Yes**	**Yes**	**Yes**
**Number of delivered messages or follow up**			**–**	**–**	**Yes**	**Yes**	**Yes**	**Yes**
**Sex**			**–**	**–**	**–**	**–**	**Yes**	**Yes**
**Age groups**			**–**	**–**	**–**	**–**	**Yes**	**Yes**
**Education level**			**–**	**–**	**–**	**–**	**Yes**	**Yes**
**Geographic area**			**–**	**–**	**–**	**–**	**Yes**	**Yes**
**Cohort**			**–**	**–**	**–**	**–**	**Yes**	**Yes**
**Observations**	**226**	**2,163**	**226**	**2,163**	**226**	**2,163**	**224**	**2,146**

Confidence intervals in parentheses.

*** p < 0.01, ** p < 0.05, * p < 0.1.

Note: The outcome variable for the 8 logistic regression models was the *vaccinated* (yes or not). The group intervention variable has two categories; those belonging to the control group and intervention (Factual, Narrative, Mixed). The word “Yes” specifies which covariates which were used to adjust the models. The sign (-) indicates that the covariate was not included in a model. Vaccination status is the vaccination status at the start of follow-up. *Not fully vaccinated*: Those who are not fully vaccinated or are eligible for a second or booster and have not received it. *Had not received any dose* are those who have not received any COVID-19 vaccine.

When analyzing each arm separately compared to the control group, the only statistically significant effect was observed in model 6 for the mixed-message group when adjusting for the message test and the number of messages (OR: 1.44; 95% CI: 1 to 2.09) (Table 3).

**Table 3:** Effect of the Intervention on new COVID-19 Vaccination by Treatment Arm.

	Model 1	Model 2	Model 3	Model 4	Model 5	Model 6	Model 7	Model 8
**Variables**	**Odds Ratio** **(95% CI)**	**Odds Ratio** **(95% CI)**	**Odds Ratio** **(95% CI)**	**Odds Ratio** **(95% CI)**	**Odds Ratio** **(95% CI)**	**Odds Ratio** **(95% CI)**	**Odds Ratio** **(95% CI)**	**Odds Ratio** **(95% CI)**
**Vaccination status at baseline**	**Had not received any dose**	**Not fully vaccinated (at least one dose)**	**Had not received any dose**	**Not fully vaccinated (at least one dose)**	**Had not received any dose**	**Not fully vaccinated (at least one dose)**	**Had not received any dose**	**Not fully vaccinated (at least one dose)**
			**Panel A: Factual vs. Control**					
**Factual *(reference group: control)***	0.42** (0.19 to 0.90)	0.84 (0.65 to 1.10)	0.52 (0.23 to 1.12)	1.03 (0.76 to 1.39)	0.87 (0.31 to 2.38)	1.20 (0.87 to 1.64)	0.87 (0.31 to 2.41)	1.13 (0.82 to 1.57)
**Observations**	**835**	**1,356**	**835**	**1,356**	**835**	**1,356**	**824**	**1,342**
			**Panel B: Mixed vs. Control**					
**Mixed *(reference group: control)***	0.46* (0.21 to 1.014)	0.94 (0.70 to 1.26)	0.61 (0.27 to 1.35)	*1.20* (0.84 to 1.72)	1.34 (0.41 to 4.38)	1.44** (1.00 to 2.09)	1.51 (0.43 to 5.26)	1.35 (0.92 to 1.98)
**Observations**	**541**	**1,062**	**541**	***1,062***	***541***	***1,062***	***538***	***1,056***
			**Panel C: Narrative vs. Control**					
**Narrative *(reference group: control)***	0.37** (0.17 to 0.81)	0.75 (0.55 to 1.02)	0.53 (0.23 to 1.19)	1.04 (0.71 to 1.53)	0.74 (0.28 to 1.92)	1.15 (0.77 to 1.70)	0.83 (0.32 to 2.13)	1.14 (0.75 to 1.72)
**Observations**	**522**	**1,043**	**522**	**1,043**	**522**	**1,043**	**520**	**1,038**
			**Model covariates**					
**Test**			Yes	Yes	Yes	Yes	Yes	Yes
**Number of delivered messages or follow up**			–	–	Yes	Yes	Yes	Yes
**Sex**			–	–	–	–	Yes	Yes
**Age groups**			–	–	–	–	Yes	Yes
**Education level**			–	–	–	–	Yes	Yes
**Geographic area**			–	–	–	–	Yes	Yes
**Cohort**			–	–	–	–	Yes	Yes

Confidence intervals in parentheses.

*** p < 0.01, ** p < 0.05, * p < 0.1.

Note: The outcome variable for the logistic regression models was the *vaccinated* (yes or not). The group intervention variable has two categories: those belonging to the control group and in each arm (Factual, Narrative, Mixed). The word “Yes” specifies which covariates which were used to adjust the models. The sign (-) indicates that the covariate was not included in a model. Vaccination status is the vaccination status at the start of follow-up. Not fully vaccinated: Those who are not fully vaccinated or are eligible for a second or booster and have not received it. Had not received any dose are those who have not received any COVID-19 vaccine.

### Survival analysis

**Fig 5:**
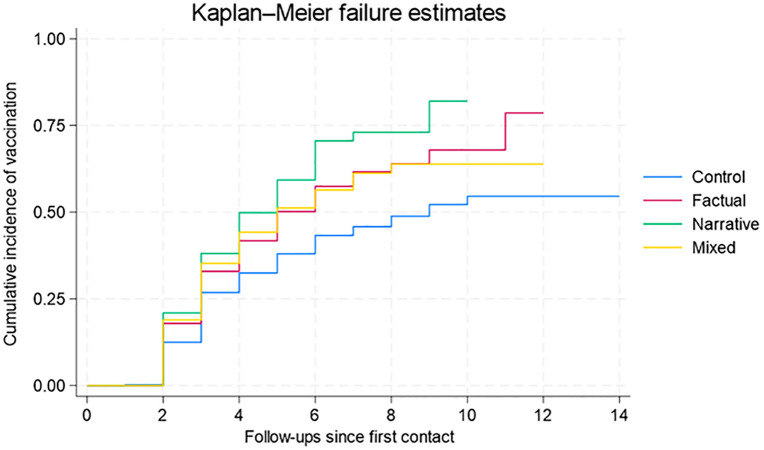
Survival analysis. Note: Kaplan–Meier curves represent the cumulative incidence of COVID-19 vaccination over follow-up contacts. Curves update only at times when vaccination events occur. The apparent early termination of curves for some intervention arms reflects the absence of additional vaccination events in later follow-up periods, with remaining participants being censored. This does not indicate lack of follow-up; participants who completed follow-up without vaccination remained under observation until the end of the study.

Fig 5 presents Kaplan–Meier failure curves showing the cumulative incidence of COVID-19 vaccination over follow-up contacts since the first contact. Across all intervention arms, vaccination occurred earlier and accumulated more rapidly than in the control group. Divergence between the intervention and control groups emerged after the first follow-up and widened steadily over time, indicating a higher and earlier probability of vaccination among participants receiving outreach messages. Among intervention arms, the mixed and narrative messages exhibited the fastest accumulation of vaccination events, while the factual arm showed a more gradual but consistently higher incidence compared with control.

Consistent with these descriptive patterns, Cox proportional hazards models —estimated over 5,775 person-follow-up contacts—demonstrated a significantly shorter time to vaccination among participants assigned to any intervention relative to control (Table 4). In the unadjusted model, exposure to any intervention was associated with a 49% higher hazard of vaccination (HR = 1.49; p < 0.001). This association remained stable after adjustment for covariates (HR = 1.50; p < 0.001).

**Table 4:** Effect Between Outreach Interventions and Time to COVID-19 Vaccination.

	Model 1	Model 2	Model 3	Model 4	Model 5	Model 6
**Variables**	**Hazard ratios** **(95% CI)** [*p value*]	**Hazard ratios** **(95% CI)** [*p value*]	**Hazard ratios** **(95% CI)** [*p value*]	**Hazard ratios** **(95% CI)** [*p value*]	**Hazard ratios** **(95% CI)** [*p value*]	**Hazard ratios** **(95% CI)** [*p value*]
**Intervention**	**Unadjusted**	**Fully** **adjusted**	**Had not received any dose** (Fully adjusted)	**Not fully vaccinated (at least one dose)** (Fully adjusted)	**Second dose uptake** **(One dose at baseline)** (Fully adjusted)	**Booster uptake** **(Fully vaccinated at baseline)** (Fully adjusted)
*(reference group: control)*	**Panel A**: Any Intervention vs Control					
	1.49 (1.22 to 1.83) [<0.001]	1.50 (1.24 to 1.83) [<0.001]	1.24 (0.63 to 2.41) [0.526]	1.53 (1.25 to 1.88) [<0.001]	1.43 (0.89 to 2.28) [0.131]	1.64 (1.30 to 2.08) [<0.001]
*(reference group: control)*	**Panel B:** Intervention Type vs Control					
**Factual group**	1.41 (1.11 to 1.78) [0.004]	1.41 (1.14 to 1.77) [0.002]	1.32 (0.66 to 2.64) [0.430]	1.43 (1.14 to 1.81) [0.002]	1.13 (0.66 to 1.94) [0.632]	1.62 (1.24 to 2.10) [<0.001]
**Mixed group**	1.71 (1.32 to 2.22) [<0.001]	1.71 (1.35 to 2.17) [<0.001]	0.89 (0.33 to 2.37) [0.820]	1.81 (1.41 to 2.32) [<0.001]	1.94 (1.17 to 3.22) [0.009]	1.79 (1.32 to 2.42) [<0.001]
**Narrative group**	1.44 (1.09 to 1.89) [0.008]	1.45 (1.11 to 1.88) [0.005]	1.47 (0.59 to 3.64) [0.398]	1.44 (1.09 to 1.90) [0.010]	1.32 (0.73 to 2.41) [0.352]	1.55 (1.12 to 2.14) [0.008]

Note: Notes: Hazard ratios estimated using Cox proportional hazards models with robust standard errors clustered at the individual level. Time was measured as the number of follow-up contacts since first outreach (5,775 person–time). Adjusted models include age group, sex, education level, geographic area, and cohort fixed effects. All hazard ratios are relative to the control group. Values in parentheses indicate 95% confidence intervals. Values in square brackets indicate p-values. Vaccination status is the vaccination status at the start of follow-up. Not fully vaccinated: Those who are not fully vaccinated or are eligible for a second or booster and have not received it. Had not received any dose are those who have not received any COVID-19 vaccine. Model 5 (Second dose uptake) restricts the risk set to individuals who had received exactly one vaccine dose at baseline; the event is receipt of the second dose, corresponding to completion of the primary vaccination series. Model 6 (Booster uptake) restricts the risk set to individuals who had completed the primary vaccination series at baseline, with or without a prior booster; the event is receipt of the next booster dose (first or second booster, depending on eligibility).

Stratified analyses by baseline vaccination status revealed heterogeneity in intervention effects. Among individuals who had not received any COVID-19 vaccine at baseline, the estimated hazard ratio was positive but not statistically significant (HR = 1.24; p = 0.526). In contrast, among participants who were not fully vaccinated at baseline, intervention exposure was associated with a substantially higher rate of vaccination over time (HR = 1.53; p < 0.001).

Further analyses examined progression along the vaccination pathway. Among individuals who had received exactly one vaccine dose at baseline, intervention exposure was not statistically significant. In contrast, among individuals who had completed the primary vaccination series at baseline, intervention exposure was associated with a significantly higher hazard of booster uptake over follow-up (HR = 1.64; p < 0.001).

When examining intervention types separately, all three message strategies were associated with faster vaccination relative to control in the fully adjusted models. The mixed intervention showed the largest point estimate (HR = 1.71; 95% CI 1.35 to 2.17; p < 0.001), followed by the narrative (HR = 1.45; 95% CI: 1.11–1.88; p = 0.005) and factual interventions (HR = 1.41 95% CI 1.14 to 1.77; p = 0.002). Stratification by baseline vaccination status indicated that these effects were primarily driven by individuals who were not fully vaccinated at study entry, whereas estimates among those with no prior doses were smaller and statistically non-significant.

Consistent with these patterns, intervention effects on second-dose uptake were generally not statistically significant across message strategies, with the exception of the mixed intervention. In contrast, intervention effects on booster uptake were larger and statistically significant for all three message strategies, with the strongest effects observed for the mixed intervention (HR = 1.79; p < 0.001), followed by factual messages (HR = 1.62; p < 0.001) and narrative messages (HR = 1.55; p = 0.008).

Across all Cox models, tests of the proportional hazards assumption based on Schoenfeld residuals did not indicate meaningful departures from proportionality (global p-values > 0.60), supporting the validity of the model specification.

### Model fit

Our logistic regression model demonstrated acceptable discrimination (AUC = 0.70). The Hosmer–Lemeshow goodness-of-fit test indicated deviation from perfect calibration (χ² = 85.9, p < 0.001). As documented in the methodological literature, the Hosmer–Lemeshow test is highly sensitive to sample size and frequently rejects adequately specified models in large samples, particularly for parsimonious specifications not designed for individual-level prediction. Because the primary objective of the analysis was causal estimation of randomized treatment effects rather than prediction, this result does not affect the validity of the intervention effect estimates.

## Discussion

This is the first study, to our knowledge, in which an IVR audio messaging intervention was used to promote COVID-19 vaccination in a middle-income country. In this randomized trial, we found that there were initially no relevant differences between control and treatment. However, when controlling for the level of understanding of the message and the number of messages delivered, any intervention type increased the odds of getting vaccinated by 40% relative to control among those who report having already received at least one COVID-19 dose at baseline compared to those who had already been vaccinated. No effects were found among those who had not received any COVID-19 vaccination at the time of the first message. Interestingly, this effect does not seem to be meaningfully influenced by the inclusion of age, sex, and education controls. The intervention type might not yield differential effects. Importantly, we found a dose-response messaging effect among those who got vaccinated, implying that level of exposure is a key determinant of the effectiveness of the intervention. In other words, our results show that the more messages, the more likely is the participants’ intention to get vaccinated.

Individuals who did not receive any vaccine at the time of the first message were not more likely to vaccinate after receiving the messages. This implies that IVR messages might have a nudge effect to promote vaccination among those already contemplating adopting the behavior, but it is not effective in changing the minds of those already unwilling to receive the vaccines. In such cases, ensuring that these messaging interventions are complemented by other strategies to improve confidence in vaccination could be crucial. Also, it is important to bear in mind that these interventions might nudge behavior on the demand side, but also the supply side barriers to access became an important barrier to access COVID-19 vaccines. Similar to previous research, we found that people willing to get vaccinated just did not get access to the vaccines [21].

The study was conducted during a period of relatively low COVID-19 severity in Colombia, following the major pandemic waves of 2020–2021 [35]. During late 2022, national surveillance data indicated low incidence, limited hospital strain, and low fatality rates, despite some localized increases in transmission. As a result, participants were making vaccination decisions—particularly regarding second doses and boosters—under conditions of reduced perceived risk and pandemic fatigue rather than acute threat. This epidemiological context is important for interpreting our findings, as intervention effects likely reflect the ability of IVR messaging to motivate vaccination in a late-pandemic setting characterized by complacency and shifting priorities rather than heightened fear of infection. There are some limitations related to this study. First, there is likely some level of self-selection bias on the choice to continue the trial or to pay attention to the content of the messages. This implies that the effects found are the average treatment effects on those more likely to internalize the message whereas those less likely to do it (i.e., individuals who were not previously vaccinated, or those who paid less attention to the message and failed the test) do not change their behavior. This might suggest that using IVR alone to promote vaccination in the midst of a pandemic works mostly as a nudge to get vaccinated, but not as an intervention that can modify the participant’s behavior. Yet, this might work as a cost-effective nudge given its relatively low cost (86.2% of those who got vaccinated did it at 4 calls, each one lasting less than three minutes at a 0.12 USD per minute cost, plus 1 USD of incentive). However, on the other hand, those in the control group ended up having higher levels of education. Since research shows that COVID-19 vaccination was more likely among more educated individuals [36–38], if our control sample is self-selected to be more educated, the treatment effects might be underestimated. In addition to this, phone ownership in Colombia is usually personal and therefore we believe we can assume that the same person is who answers the phone. However, the way the IVR works does not allow us to assess whether the person answering the phone every single time is the same.

Second, since we had to carry out the control arm to capture the effects of the messaging strategy, it is possible that those in the control arm were nudged to get vaccinated just by getting the call without the message, implying that our results reveal the effects of the messages themselves but the actual call with the messages might have even greater effects.

Third, the depth of personalization is still limited compared to human interactions. Standardized messages may not account for individual differences in cultural background, language proficiency, or varying health literacy levels, which could affect the intervention’s effect and tailoring strategies are encouraged to be tested in further studies, especially in countries with a diverse cultural and ethnic background. To this end, we conducted preliminary work to better understand the cultural factors that might make the interventions more effective and the co-design of the interventions is described in a previous publication [32].

Fourth, naturally, the change in vaccination status is self-reported, and the treatment intervention may have a differential effect on self-reporting rather than actual change of vaccination status due to social desirability bias. In addition to this, we don’t have population-level data by age group with which we can assess the level of self-selection bias in our sample due to this variable.

Fifth, follow-up is also a limitation: Future studies should incorporate time as a variable by extending the follow-up duration (e.g., cohort studies) in order to capture longer follow-up periods and allow for the identification of thresholds in effect duration. This would allow for the capture not only the immediate effects of the intervention but also its long-term sustainability, potential delayed impacts, and the evolution of behavioral changes over time, especially given that there might have been supply factors limiting the ability for respondents to vaccinate during the trial.

## Conclusion

This audio messaging intervention using IVR appears to have been an effective and possibly a cost-effective [39] approach to support COVID-19 vaccination uptake in Colombia. IVR messaging presents important opportunities in emergency conditions, where it is easier to reach populations in remote or underserved areas, given that most individuals have access to a telephone network. This would allow interventions to be scaled up in a timely manner and allows to handle large volumes of participants simultaneously without additional human resources, making them efficient for high-volume interventions. We believe this strategy should be further examined to inform potential use by decision-makers in efforts to increase the vaccination rate or promote other healthy behaviors, particularly in sanitary emergencies in LMICs.

### Contributions to the literature

- Mobile phone message interventions through interactive voice response (IVR) can motivate vaccination in emergency conditions among those already convinced of the importance of getting vaccinated. Mobile phone messages serve as a nudge for those already convinced of the importance of vaccination but are less likely to change the minds of those previously hesitant to be vaccinated.
- Understanding the content of the messages is a key factor in the success of the interactive voice response messaging intervention.
- IVR messaging shows a clear dose–response relationship, the greater the number of messages received, the higher the participants’ intention to get vaccinated.

## Supporting information

S1 Table Factual and narrative co-designed messages. (DOCX)

S1 Text Study protocol. (PDF)

S1 Checklist CONSORT 2025 checklist. (DOCX)

S2 Checklist Inclusivity in global research checklist. (DOCX)
