# Supplementary material for: The effect of mobile phone messaging to support COVID-19 vaccination in Colombia: A randomized controlled clinical trial
Source: PLOS Glob Public Health. 2026 Jun 11;6(6):e0006387. doi: 10.1371/journal.pgph.0006387 (PMC13257970; doi:10.1371/journal.pgph.0006387)
Supplement: S1 Table — (DOCX) [file pgph.0006387.s001.docx]

**S1 Table:** Factual and narrative co-designed messages (obtained from Aya et al[31])

| **No.** | **Structure of the message** | | **Message categories and topics in English (translated)** | | **Message categories and topics in Spanish (original)** | **Sender** | | |  |  |
| --- | --- | --- | --- | --- | --- | --- | --- | --- | --- | --- |
| ***Factual Messages*** | | | | | | | | | |  |
| **1** | **Vaccination and infection** | | | | | | | | |  |
|  | Whole message | | Did you know that getting vaccinated against COVID-19 reduces the risk of developing severe symptoms, needing hospitalization, and dying?   Vaccines against COVID-19, as for other diseases, help so that if you do get sick, the symptoms are milder than if you did not have the vaccine.  For example, according to data from the Colombian Ministry of Health, people who have been vaccinated against COVID-19 have three times less risk of hospitalization than unvaccinated people.   If you have not yet been vaccinated or are missing a dose, remember to do so as soon as possible. | | ¿Sabía usted que vacunarse contra COVID-19 reduce el riesgo de desarrollar síntomas graves, de necesitar hospitalización y de morir?   Las vacunas contra COVID-19, al igual que para otras enfermedades, ayudan a que si te enfermas, los síntomas sean más suaves que si no tuvieras la vacuna.   Por ejemplo, de acuerdo a datos del Ministerio de Salud de Colombia, las personas que han sido vacunadas contra COVID-19, tienen tres veces menos riesgo de hospitalización que las personas no vacunadas.   Si aún no se ha vacunado o le falta alguna dosis, recuerde hacerlo lo antes posible. | Woman journalist’s voice (Voice B) | |  |  |  |
| **2** | **Vaccine effectiveness** | | | | | | | | |  |
|  | Whole message | | | Did you know that as of June 2022, eight out of ten Colombians had received at least one dose of the COVID-19 vaccine and that, thanks to this, more than 36,000 lives have been saved?   In addition, more than half of the people vaccinated against COVID-19 did not need hospitalization when they became infected.   If you have not yet been vaccinated, remember to do it soon because we are in a new peak of COVID-19. If you just got COVID-19, remember to wait 30 days to get vaccinated.   It is necessary to get vaccinated because the defenses that your body generated during that infection will not be enough to fight a new attack of the virus. | ¿Sabía usted que a junio de 2022, 8 de cada 10 colombianos se había aplicado al menos una dosis de la vacuna contra COVID-19 y que, gracias a esta, se han salvado más de 36,000 vidas?   Además, más de la mitad de las personas vacunadas contra COVID-19 no necesitaron hospitalización cuando se contagiaron.   Si aún no se ha vacunado, recuerde hacerlo pronto porque estamos en un nuevo pico de COVI-19. Si le acaba de dar COVID-19, recuerde esperar 30 días para vacunarse.   Es necesario vacunarse porque las defensas que generó su cuerpo durante esa infección, no serán suficientes para combatir un nuevo ataque del virus. | | Woman journalist’s voice (Voice B) | | | |
| **3** | **Vaccine development process** | | | | | | | | |  |
|  | Whole message | | | Did you know that COVID-19 vaccines were developed following rigorous approval and safety monitoring processes around the world?   It was faster to create the vaccine against COVID-19 than for other diseases because we have more technology, because there were already scientific studies to design vaccines against other similar diseases, and because this time the scientific community around the world worked collaboratively. Thanks to all this, as of June 2022, more than 42 million people in Colombia have been vaccinated with at least one dose of the vaccine against COVID-19.   Vaccinating ourselves protects us all. If you have not yet been vaccinated or are missing a dose, remember to do so as soon as possible. | ¿Sabía usted que las vacunas contra COVID-19 se desarrollaron siguiendo rigurosos procesos de aprobación y monitoreo de su seguridad en todo el mundo?   Fue más rápido crear la vacuna contra COVID-19 que para otras enfermedades porque tenemos más tecnología, porque ya había estudios científicos para diseñar vacunas contra otras enfermedades parecidas y, porque esta vez la comunidad científica de todo el mundo trabajó colaborativamente. Gracias a todo esto, a junio de 2022 más de 42 millones de personas en Colombia han sido vacunadas con al menos una dosis de la vacuna contra COVID-19.   Vacunándonos nos protegemos todos. Si aún no se ha vacunado o le falta alguna dosis, recuerde hacerlo lo antes posible. | | Woman journalist’s voice (Voice B) | | | |
| **4** | **Vaccine ingredients** | | | | | | | | |  |
|  | Whole message | | | Have you ever wondered what COVID-19 vaccines are made of?   Regardless of the brand, because they all work and are safe, these vaccines do not contain eggs, gluten, or preservatives. They also do not have metals, plastics, and electronic equipment. Vaccines teach the body to build defenses against future attacks by the virus.   If you have not yet been vaccinated against COVID-19 or are missing a dose, remember to do so as soon as possible. | ¿Alguna vez se ha preguntado de qué están hechas las vacunas contra COVID-19?   Sin importar la marca, porque todas sirven y son seguras, éstas vacunas no contienen huevos, gluten, ni preservativos. Tampoco tienen metales, plásticos, ni equipos electrónicos. Las vacunas le enseñan al cuerpo a generar defensas contra futuros ataques del virus.   Si aún no se ha vacunado contra COVID-19 o le falta alguna dosis, recuerde hacerlo lo antes posible. | | Woman journalist’s voice (Voice B) | | | |
| **5** | **Security and adverse effects** | | | | | | | | |  |
|  | Whole message | | | Did you know that COVID-19 vaccines have prevented three out of five people infected with COVID-19 from being hospitalized?   But, getting vaccinated, like any change that is made in the body, can have an effect.   In studies conducted to ensure that the vaccines were safe, involving thousands of people, it was found that some people reported temporary effects of the vaccines, including temporary pain in the arm where the vaccine was given, some tiredness, fever or nausea, and in some women, alterations in menstruation.  Remember that the possible discomfort caused by the vaccine is temporary and that the benefits of the vaccine are even greater. It could save your life.  If you have not yet been vaccinated or are missing a dose, remember to do so as soon as possible. | ¿Sabía usted que las vacunas contra COVID-19 han evitado que tres de cada cinco personas infectadas con COVID-19 sean hospitalizadas?   Pero, vacunarse, como cualquier cambio que se hace en el cuerpo puede tener un efecto.   En los estudios que se hicieron para garantizar que las vacunas fueran seguras, donde participaron miles de personas, se encontró que algunas personas reportaron efectos temporales de las vacunas entre estos, dolor transitorio en el brazo en el que se aplicó la vacuna, un poco de cansancio, fiebre o náuseas y en algunas mujeres, alteraciones en la menstruación.  Recuerde que los posibles malestares causados por la vacuna son temporales y que los beneficios de la vacuna son aún mayores. Podría salvarle la vida.  Si aún no se ha vacunado o le falta alguna dosis, recuerde hacerlo lo antes posible. | | Woman journalist’s voice (Voice B) | | | |
| **6** | **Booster dose** | | | | | | | | |  |
|  | Whole message | | | Did you know that COVID-19 booster vaccines help maintain protection against severe symptoms of the disease?   When we get booster doses, we increase our body's immunity and its ability to fight a new attack from the virus. This is important because, as with other vaccines, the protection of vaccines against COVID-19 decreases as time passes and the disease changes.   Remember that the first booster dose is available to everyone over 18 years of age and is given four months after completing the vaccination schedule.   If you are missing the booster dose, remember to get it as soon as possible. | ¿Sabía usted que las vacunas de refuerzo contra COVID-19, ayudan a mantener la protección contra los síntomas graves de la enfermedad?   Cuando nos aplicamos las dosis de refuerzo, incrementamos la inmunidad de nuestro cuerpo y su capacidad para combatir un nuevo ataque del virus. Esto es importante porque, así como sucede con otras vacunas, la protección de las vacunas contra COVID-19 va disminuyendo en la medida que pasa el tiempo y la enfermedad va cambiando.   Recuerde que la primera dosis de refuerzo está disponible para todas las personas mayores de 18 años y, se aplica cuatro meses después de haber completado el esquema de vacunación.   Si le falta la dosis de refuerzo, recuerde hacerlo lo antes posible. | | Woman journalist’s voice (Voice B) | | | |
| **7** | **Family protection and care** | | | | | | | | |  |
|  | Whole message | | | Did you know that studies conducted in Colombia and other countries have found that vaccines against COVID-19 help to protect adults, adolescents, boys and girls with whom we live?   Thanks to these vaccines, more than 36,000 lives were saved last year in Colombia.   Vaccinating ourselves protects us all. If you have not yet been vaccinated or are missing a dose, remember to do so as soon as possible. | ¿Sabía usted que estudios realizados en Colombia y otros países han encontrado que las vacunas contra COVID-19 ayudan a proteger a los adultos, adolescentes, niños y niñas con quienes convivimos?   Gracias a estas vacunas, el año pasado se salvaron más de 36,000 vidas en Colombia.   Vacunándonos nos protegemos todos. Si aún no se ha vacunado o le falta alguna dosis, recuerde hacerlo lo antes posible. | | Woman journalist’s voice (Voice B) | | | |
| ***Narrative Messages*** | | | | | | | | | |  |
| **8** | **Vaccination and infection** | | | | | | | | |  |
|  | Start and contextualization | | | Hearing is Believing. We interviewed people from all over Colombia, and do you know what they told us about vaccination against COVID-19? | Oír para creer, entrevistamos a personas de todo Colombia y, ¿saben qué nos contaron sobre la vacunación contra COVID-19? | | Young man’ voice  (Voice B) | | | |
|  | Core message | | | “At the beginning of the pandemic it was a bit difficult. One was used to a work routine, and social life and, well, suddenly, for health reasons, everything changed. But precisely, the vaccine is to ensure that, if you do get infected, it won't hit you so hard.” | "Al principio de la pandemia fue un poco difícil, uno venía acostumbrado a una rutina de trabajo, vida social y, pues, de repente, por cuestiones de salud, todo cambió. Pero, precisamente, la vacuna es para que, si llegado el caso te llegas a contagiar, no te pegue tan fuerte". | | Woman’s voice  (Not tested) | | | |
|  | Ending with call to action | | | Vaccines reduce the risk of developing severe symptoms. If you have not yet been vaccinated or are missing a dose, remember to visit the nearest vaccination center. | Las vacunas reducen el riesgo de desarrollar síntomas graves. Si aún no te has vacunado o te falta alguna dosis, recuerda visitar el punto de vacunación más cercano. | | Young man’s voice  (Voice B) | | | |
| **9** | **Vaccine effectiveness and vaccination / infection** | | | | | | | | |  |
|  | Start and contextualization | | | Hearing is Believing. We interviewed people from all over Colombia, and do you know what they told us about vaccination against COVID-19? | Oír para creer, entrevistamos a personas de todo Colombia y, ¿saben qué nos contaron sobre la vacunación contra COVID-19? | | Woman journalist’s voice | | | |
|  | Core message | | | "I got COVID twice and it left me with sequelae: with many respiratory problems and now I am in treatment. So, in order to get vaccinated I had to ask my doctors and they told me that there was no problem, so I got vaccinated with two dose and I am waiting for the third one. And I tell you that, to my surprise, I thought that due to my sequelae I was going to have some reaction from the vaccine and hey, no! I didn't get me anything." | "A mí me dio dos veces COVID y me dejó secuelas: con muchos problemas respiratorios y ahora estoy en tratamiento. Entonces, para poder vacunarme tuve que preguntarles a mis médicos y ellos me dijeron que no había ningún problema, así que me vacuné con dos dosis y estoy pendiente de la tercera. Y le cuento que, para mi sorpresa, pensé que por mis secuelas iba a tener alguna reacción por la vacuna y ¡oiga, no! no me dio nada." | | Older man’ voice  (Not tested) | | | |
|  | Ending with call to action | | | Even if you have been infected with COVID-19 you can also get vaccinated. If you have not yet been vaccinated or are missing a dose, remember to visit the nearest vaccination center. | Así te hayas enfermado de COVID-19 también puedes vacunarte. Si aún no te has vacunado o te falta alguna dosis, recuerda visitar el punto de vacunación más cercano. | | Woman journalist’s voice | | | |
| **10** | **Vaccine development process** | | | | | | | | |  |
|  | Start and contextualization | | | Hearing is Believing. We interviewed people from all over Colombia, and do you know what they told us about vaccination against COVID-19? | Oír para creer, entrevistamos a personas de todo Colombia y, ¿saben qué nos contaron sobre la vacunación contra COVID-19? | | Young man’s voice  (Voice B) | | | |
|  | Core message | | | “Vaccination, for me, is something very important. It really is that with the crisis that occurred, it was very impressive that they released the vaccines so quickly! When I found out about the vaccines, I felt happy, because, fortunately, despite working in a hospital, I did not get infected. With the vaccine, I became more comfortable at work. I am a surgical instrument assistant, and I have always believed in science, because it is  available to us, to act quickly in these situations". | "La vacunación, para mí, es algo muy importante. Realmente es que con la crisis que hubo, ¡fue muy impresionante que hayan sacado tan rápido las vacunas! Cuando me enteré de las vacunas, me sentí feliz, porque, afortunadamente, a pesar de estar trabajando en un hospital, no me contagié. Ya con la vacuna estaba más tranquila en mi trabajo. Soy instrumentadora quirúrgica, y siempre he creído en la ciencia, porque ella está disponible para nosotros, para actuar rápido ante estas situaciones." | | Woman’s voice  (Not tested) | | | |
|  | Ending with call to action | | | Vaccines work. If you have not yet been vaccinated or are missing a dose, remember to visit the nearest vaccination center. | Las vacunas funcionan. Si aún no te has vacunado o te falta alguna dosis, recuerda visitar el punto de vacunación más cercano. | | Young man’s voice  (Voice B) | | | |
| **11** |  | **Protection to society / social responsibility** | | | | | | | |  |
|  | Start and contextualization | | | Hearing is Believing. We interviewed people from all over Colombia, and do you know what they told us about vaccination against COVID-19? | Oír para creer, entrevistamos a personas de todo Colombia y, ¿saben qué nos contaron sobre la vacunación contra COVID-19? | | Young man’s voice  (Voice B) | | | |
|  | Core message | | | "My decision was always to get vaccinated for prevention, health, respect to my family and at a societal level, because I believe that it is a matter of social responsibility that everyone gets vaccinated; I believe that we all have a risk, we do not know how the virus will react in each person and well, I believe that we should be motivated by the love to our family and to ourselves, and that, in my case, I have to be healthy to protect them in some way; so I already have the three doses of the vaccine and all is going well, I didn’t have any type of reaction in any of the three doses. My family is also vaccinated and none of them had adverse effects". | "Mi decisión siempre fue vacunarme por prevención, por salud, por respeto a mi familia y a nivel social, porque creo que es algo de responsabilidad social, que todos debemos estar vacunados; considero que todos tenemos un riesgo, no sabemos cómo va a reaccionar el virus en cada persona y bueno, creo que debería motivarnos, principalmente, el amor a nuestra familia y a nosotros mismos, y por eso, en mi caso, debo estar bien y protegerlos de cierta manera; entonces, ya tengo las tres dosis de la vacuna y súper bien, no tuve ningún tipo de reacción en ninguna de las tres dosis. También mi familia está vacunada y ninguno tuvo síntomas". | | Young man’s voice  (Voice B) | | | |
|  | Ending with call to action | | | Taking care of ourselves is everyone's responsibility. If you have not yet been vaccinated or you are missing a dose, remember to visit the nearest vaccination center. | Cuidarnos es responsabilidad de todos. Si aún no te has vacunado o te falta alguna dosis, recuerda visitar el punto de vacunación más cercano. | | Young man’s voice  (Voice B) | | | |
| **12** |  | **Security and adverse effects** | | | | | | | |  |
|  | Start and contextualization | | | Hearing is Believing. We interviewed people from all over Colombia, and do you know what they told us about vaccination against COVID-19? | Oír para creer, entrevistamos a personas de todo Colombia y, ¿saben qué nos contaron sobre la vacunación contra COVID-19? | | Young man’s voice  (Voice B) | | | |
|  | Core message | | | "Right now, I am unemployed because due to the pandemic the school where I worked had to close. The pandemic changed our lives 100%... and vaccination was a way to survive this virus because we knew that if it hit us, we could survive with the vaccine, so, for everyone's wellbeing, in my family we are all vaccinated. In my case, I have all the doses and the booster. I was afraid to get this last one because of all the comments I heard about that vaccine, but no, it was normal. It went super well for my husband and me who got it." | "En este momento estoy desempleada porque debido a la pandemia el colegio donde yo trabajaba tuvo que cerrar. La pandemia nos cambió la vida 100%... y la vacunación fue una manera de sobrevivir a este virus, porque sabíamos que, si nos daba, podríamos sobrevivir con la vacuna, entonces, por el bienestar de todos, en mi familia todos estamos vacunados. En mi caso, tengo todas las dosis y la de refuerzo. Tuve miedo de ponerme esta última por todos los comentarios que escuchaba de esa vacuna, pero no, normal. Nos fue super bien a mi esposo y a mí que nos la pusimos." | | Woman’s voice  (Not tested) | | | |
|  | Ending with call to action | | | Vaccines are safe. If you have not yet been vaccinated or are missing a dose, remember to visit the nearest vaccination center. | Las vacunas son seguras. Si aún no te has vacunado o te falta alguna dosis, recuerda visitar el punto de vacunación más cercano. | | Young man’s voice  (Voice B) | | | |
| **13** |  | **Freedom to choose and vaccine security** | | | | | | | |  |
|  | Start and contextualization | | | Hearing is Believing. We interviewed people from all over Colombia, and do you know what they told us about vaccination against COVID-19? | Oír para creer, entrevistamos a personas de todo Colombia y, ¿saben qué nos contaron sobre la vacunación contra COVID-19? | | Woman journalist’s voice | | | |
|  | Core message | | | “The vaccine has worked well, after the vaccination, the number of deaths due to COVID-19 decreased. So, I believe that people should get vaccinated, well, to be safer, to live more peacefully, and continue living everyday life; and for that, we must do our part.” | "La vacuna ha servido mucho, después de la vacunación, disminuyó el número de muertes por COVID-19. Entonces, creo yo que la gente debería vacunarse, pues, para estar más seguros, para vivir más tranquilos y seguir viviendo la cotidianidad; y para eso tenemos que poner de nuestra parte." | | Young man’s voice  (Voice B) | | | |
|  | Ending with call to action | | | By vaccinating against COVID-19 we are safer. If you have not yet been vaccinated or are missing a dose, remember to visit the nearest vaccination center. | Vacunándonos contra COVID-19 estamos más seguros. Si aún no te has vacunado o te falta alguna dosis, recuerda visitar el punto de vacunación más cercano. | | Woman journalist’s voice | | | |
| **14** |  | **Family protection and care** | | | | | | | |  |
|  | Start and contextualization | | | Hearing is Believing. We interviewed people from all over Colombia, and do you know what they told us about vaccination against COVID-19? | Oír para creer, entrevistamos a personas de todo Colombia y, ¿saben qué nos contaron sobre la vacunación contra COVID-19? | | Young man’s voice  (Voice B) | | | |
|  | Core message | | | "My five-year-old son is already vaccinated too, well, I didn't want him to get sick either, and since he was going to school and going out with his friends, that’s why. I love my children so much! I adore them and would give my life for them, and that's why I got vaccinated, because maybe one day I go out, get infected, I come and hug them or kiss them and spread the virus to them... I had to do it for them.” | "Mi hijo de cinco añitos ya está vacunado también, pues, porque tampoco quería que él se enfermara y como estaba yendo al colegio y salía con amiguitos, entonces, por eso. ¡Yo quiero tanto a mis hijos! Los adoro y daría la vida por ellos, y por eso me vacuné, porque que tal un día salga, me contagie, yo llegue y los abrace o los bese y les pase el virus… yo tenía que hacerlo por ellos." | | Woman’s voice  (Not tested) | | | |
|  | Ending with call to action | | | Protect yourself and your family. If you have not yet been vaccinated or are missing a dose, remember to visit the nearest vaccination center. | Protégete y protege a tu familia. Si aún no te has vacunado o te falta alguna dosis, recuerda visitar el punto de vacunación más cercano. | | Young man's voice  (Voice B) | | | |
