# Supplementary material for: The effect of mobile phone messaging to support COVID-19 vaccination in Colombia: A randomized controlled clinical trial
Source: PLOS Glob Public Health. 2026 Jun 11;6(6):e0006387. doi: 10.1371/journal.pgph.0006387 (PMC13257970; doi:10.1371/journal.pgph.0006387)
Supplement: S1 Text — (PDF) [file pgph.0006387.s002.pdf]

JOHNS HOPKINS BLOOMBERG SCHOOL of PUBLIC HEALTH PHIRST

Date: Friday, May 23, 2025 11:26:32 AM Print Close

View: AP: Study Description and Study Team

Study Team and Study Description

1.0 * Study Title (formal title of your study):
Digital Applications to Monitor Novel Coronavirus Disease and Response in Colombia - Surveillance

2.0 * Provide a short description of your study (1-3 sentences). **If your project is a planning phase activity**, confirm that you will not involve human subjects and estimate how long the planning phase activity will take.
We will assist the Government of Colombia on its COVID-19 surveillance program and on its COVID-19 National Vaccination Plan.

1) We will deploy monthly syndromic surveys for COVID-19 monitoring using mobile phone technology (Mobile phone surveys or MPS) in municipalities considered critical by this entity due to 1) low seroprevalence, 2) high infection rate, or 3) high proportion of vulnerable population. Syndromic surveys will be deployed using Interactive Voice Response (IVR) and will allow us to support the country to detect changes in the prevalence of respiratory symptoms in these critical municipalities, allowing the Ministry of Health to act quickly on COVID-19 outbreaks, particularly with the upcoming Delta variant-driven fourth wave.

2) We will collect data from a convenience sample of 10-20 individuals declaring not being vaccinated and that consent to be recontacted in the initial call. We will carry out a brief interview and ask them about their perceptions and social representations about COVID-19 vaccines. This effort will assist on the design of the messages in the next activity.

3) Individuals who have not been partially or fully vaccinated, and who consent to being recontacted will be called back and will be invited to participate in the messaging strategy. In this one, respondents who consent to participate will be randomized in two to three different arms and based on the qualitative interviews, they will be presented with different messages about COVID-19 vaccines. Individuals will receive messages and be followed up monthly for three months to assess the best messaging strategy, so the government can deploy it to increase vaccination coverage.

3.0 * Do you intend to share the results of your project by publication, presentation, or other public dissemination beyond your sponsor and collaborators? Note: For the purposes of this question, presentation within the School is considered an academic activity, not publication or dissemination.

● **Yes** ○ No

STUDY TEAM

All investigators and study team members who will interact with participants or access identifiable personal information/biospecimens must have human subjects research ethics training. Hopkins investigators and study staff must register in PHIRST. Co-investigators from other institutions who need access to study documents in PHIRST must be registered in PHIRST. Co-investigators from other institutions who do not need access to study documents in PHIRST do not need to register in PHIRST but must be listed below and must upload their human subjects research ethics training documentation.

4.0 * Principal Investigator: Select the JHSPH faculty member (or other approved PI) responsible for this institution's role in this research activity. This person must be registered in the PHIRST system.
Andres Ignacio Vecino Ortiz

4.1 * Does the principal investigator (or their spouse, domestic partner, or dependent children) have a financial interest or fiduciary relationship with the sponsor or manufacturer/owner of products related to this research? This applies to current interests/relationships and those within the past 12 months.

(A financial interest or fiduciary relationship is one that could reasonably appear to be related to the proposed research. Examples include, but are not limited to: (1) consulting, advisory, or speaking services (paid & unpaid); (2) fiduciary role (e.g., officer, member of board of directors); (3) remuneration/income for services (e.g., honoraria, consulting fees); (4) ownership of equity, including

stock, stock options, etc.; and/or (5) entitlement to royalty.

All conflicted individuals must disclose potential conflicts of interest to the Office of Outside Interests (OOI) via eDisclose before this application can be approved.

○ Yes ● **No**

4.2 * PI Faculty Title:
Associate Scientist

5.0 Co-Investigator(s): List the names of PHIRST registered collaborating investigators who are "engaged" in human subjects research, (e.g., are interacting/intervening with participants, obtaining informed consent, or accessing/using individually identifiable data/specimens). Other co-investigators may be mentioned in publications, but they do not need to be listed here.

	Last Name	First Name	Department	E-Mail	Profile	COI
View	Ali	Joe	International Health	jali@jhu.edu	Joe Ali	no
View	Gibson	Dustin	International Health	dgibso28@jhu.edu	Dustin Gibson	no
View	Labrique	Alain	International Health	alabriqu@gmail.com	Alain Labrique	no
View	Maniar	Vidhi	Health, Behavior & Society	vmaniar1@jh.edu	Vidhi Maniar	no
View	Trujillo	Antonio	International Health	atrujill@jhsph.edu	Antonio Trujillo	no

6.0 Co-Investigator(s) not registered in PHIRST: List each co-investigator from other institutions who will interact with study participants or access identifiable personal information, but who do not need access to study documents in PHIRST. These individuals do not need to register in PHIRST, but must upload documentation of their human subjects research ethics training and an Investigator's Agreement form. NOTE: This does not constitute a Reliance Agreement with the BSPH IRB for non-JHU study team members. Investigators from other institutions must obtain approval from their own IRB/Ethics Committee, and/or request a Reliance Agreement via question 13.0 below.

Last Name	First Name	Agreement Form	HST Cert	GCP Cert	HIPAA Cert

There are no items to display

7.0 Student Investigator(s): List all JHU graduate "student investigators" on the study. A "student investigator" is one who undertakes a research project in partial fulfillment of a degree program or other educational requirements under the supervision of a faculty PI. A student who is working on a study (as an employee or otherwise) is a "Study Team Member" and should be listed under the "Other Study Team Members" section below.

Last Name	First Name	Department	Email	Profile	COI

There are no items to display

8.0 * Study Contact: Select the name of the person that should receive all PHIRST correspondence (PI or other) in addition to the PI.
Vidhi Maniar

8.1 * Does the study contact (or their spouse, domestic partner, or dependent children) have a financial interest or fiduciary relationship with the sponsor or manufacturer/owner of products related to this research? This applies to current interests/relationships and those within the past 12 months.

(A financial interest or fiduciary relationship is one that could reasonably appear to be related to the proposed research. Examples include, but are not limited to: (1) consulting, advisory, or speaking services (paid & unpaid); (2) fiduciary role (e.g., officer, member of board of directors); (3) remuneration/income for services (e.g., honoraria, consulting fees); (4) ownership of equity, including stock, stock options, etc.;

and/or (5) entitlement to royalty.

All conflicted individuals must disclose potential conflicts of interest to the Office of Outside Interests (OOI) via eDisclose before this application can be approved.

○ Yes ● **No**

9.0 Other Study Team Members: List the name of PHIRST registered study team members who are essential to study operations and are "engaged" in human subjects research, e.g. are interacting/intervening with participants, obtaining informed consent, or accessing/using individually identifiable data/specimens. These could include Project Directors, Research Coordinators, Data Administrators, Biospecimen Repository Managers, etc. Describe in your research plan how other personnel who are not listed in PHIRST and who may also be "engaged" in human subjects research will be trained in human subjects research ethics. NOTE: This does not constitute a Reliance Agreement with the BSPH IRB for non-JHU study team members. Investigators from other institutions must obtain approval from their own IRB/Ethics Committee and/or request a Reliance Agreement via question 13.0 below.

Last Name	First Name	Department	E-Mail	Profile	COI

There are no items to display

10.0 * Is this a "planning phase application", e.g., are you seeking IRB approval for developmental or formative work that will not involve any interaction with study participants?

○ Yes ● **No**

10.1 * Does this study have an associated approved planning phase application?

○ Yes ○ No

11.0 * Is your study under review by the Western IRB (WIRB)?

○ Yes ● **No**

11.1 *

11.2 *

11.3

12.0 * Does your study meet a definition of "clinical trial" , i.e., a prospective assignment of one or more interventions to evaluate the effects of those interventions on health-related biomedical or behavioral outcomes?

○ Yes ● **No**

12.1

12.2 * By checking the box, you confirm that all investigators and study staff on this study will complete GCP training (see: http://grants.nih.gov/grants/guide/notice-files/NOT-OD-16-148.html).

Confirm

13.0 * Do you want the BSPH IRB to rely on a U.S. based, non-Hopkins external sIRB for non-exempt research, or accept an Exempt Determination from a U.S. based, non-Hopkins external IRB?

○ Yes ● **No**

14.0 *Does your study involve any of the following? Select all that apply.

None of these are involved

14.1 *

If yes, please upload all necessary documents on the Miscellaneous Documents page in the "Other Documents" section

View: AP: Research Data Description (Real)

Research Data Description

1.0 * Does the proposed research involve obtaining (i.e., collecting and/or receiving) information about and/or from **living** individuals?

(●) **Yes** ( ) No

2.0 * Will your research activity involve the **secondary** analysis of existing data from records, research datasets, and/or biospecimens (e.g., exists at the time you submit your application) about living individuals?

( ) Yes (●) **No**

3.0 * Will your study involve **new collection** of information from/or about individuals?

(●) **Yes** ( ) No

3.1 * Is the new data collection for this research study limited to information from Key Informants (click on blue ? for more information).

( ) Yes (●) **No**

3.2 * Will you only collect data using an anonymous online survey/questionnaire?

( ) Yes (●) **No**

4.0 * Will the identifiable data you propose to access for research purposes (including recruitment of patients inside a clinic or hospital), whether "existing" or newly collected, come from a U.S. based healthcare provider or billing organization (e.g., is it Protected Health Information (PHI) from a U.S. "covered entity")? Medicare claims data falls into this category.

( ) Yes (●) **No**

4.1 * Will the PHI involve U.S. based clinical records and information about more than 500 individuals?

( ) Yes ( ) No

4.2 * Will the research activity (including recruitment) be conducted through a Hopkins clinical facility (with Hopkins patients, Hopkins resources, Hopkins data, or a Hopkins or JHHC administered health plan) or the Baltimore City Health Department (BCHD)?

( ) Yes ( ) No

4.3 * Will the research activity (including requirement) be conducted through a NON-HOPKINS, U.S. based clinical healthcare provider, clinical data registry, or health billing organization (e.g., University of Maryland hospital, public health clinics, private physician's office, state cancer registry, etc.)?

( ) Yes ( ) No

5.0 * Will identifiable data come from a non-U.S. based health care provider (e.g. a foreign research site) and brought to a U.S. based covered entity (such as Johns Hopkins School of Medicine)?

( ) Yes (●) **No**

6.0 * Will you conduct the research in a public school, recreation center, market, foreign health clinic, Native American reservation, or other site that requires local permission/approval for access?

○ Yes ● **No**

7.0 Are the data you will collect or access for research purposes sensitive such that its disclosure outside the study could expose the participants to harm, such as:

- social ostracization
- threat to legal status
- significant psychological/emotional distress
- economic loss or threat to employment, or
- civil/criminal liability

○ Yes ● **No**

8.0 * Does your study have Certificate of Confidentiality Protections?

○ Yes ● **No**

9.0 * Select one or more of the following that best describes your study:

- [ ] Secondary Data Analysis of existing dataset (including clinical data, research data, data from outside sources)
- [x] New Data Collection
- [x] Program Project (assessment, evaluation, implementation, scale-up, etc) in collaboration with public health authority
- [ ] Biospecimen or Data Repository
- [ ] Data Coordinating Center (no enrollment at JHSPH site)
- [ ] Data Coordinating Center (with enrollment at JHSPH supervised site)
- [ ] Other (specify data from outside sources, and/or analysis of existing biospecimens)

View: AP: Study Population

Study Population

1.0 * List the countries where the data will be collected. Select all that apply.

Country
Colombia

2.0 *Check the population(s) that meet your study's inclusion criteria:

Children

- [ ] Infants (0-12 months)
- [ ] Children (1-12 yrs old)
- [ ] Adolescents (13-17 yrs old)
- [ ] Pregnant Adolescents
- [ ] Children in Foster Care or Outside of Family Care

Adults

- [x] Women
- [ ] Pregnant Women
- [x] Men
- [ ] Non-Binary
- [ ] Adults with Cognitive Impairment
- [ ] Baltimore City Employees or BCHD clients
- [ ] JHU/JHH Employees or Students (all divisions)
- [ ] Non-English Speakers, where English is the primary language
- [ ] U.S. Military Personnel
- [ ] Prisoners (any gov't imposed limitation on freedom)

3.0 * Will the study include subjects from an existing cohort or population?
○ Yes ● **No**

3.1 Name of Cohort/Population:

3.2 Cohort Study IRB Number:

3.3 Cohort Study Title:

3.4

View: AP: Research Plan

Research Plan

The JHSPH IRB website includes two research templates for you to consider: Research Plan for Secondary Analysis of Data/Specimens, and Research Plan for New Data Collection (and Secondary Data Analysis, if applicable). Visit the Research Plan site to download and complete the template most appropriate for your study, then upload it here.

1.0 *Upload your **Research Plan Clean Version**:

	Title	Owner	Modified Date
View	Public Health Surveillance determination form(0.03)	Andres Ignacio Vecino Ortiz	1/21/2022 11:56 AM

When you upload new versions of the clean or tracked research plan, you will be given the option of adding a title to the document. If you do not provide a title, the electronic file name of the document will be the default title. The PHIRST system will note the number of versions you upload.

2.0 Upload your **Research Plan Tracked Version**:

Title	Owner	Modified Date

There are no items to display

View: AP: Collaborations Funding and COI

Collaborations and Funding

1.0 * Will the research involve collaboration with a non-Hopkins entity?
● **Yes** ○ No

1.1 * Enter information about the collaborating institutions(s):

	Name of Organization
View	Pontificia Universidad Javeriana

2.0 * Will you be obtaining IRB or REC (Research Ethics Committee) approval from other than the JHSPH IRB?
Yes

2.1 * Enter information about the other IRB(s) or REC(s). Provide the FWA number if there is one; check https://ohrp.cit.nih.gov/search/fwasearch.aspx?styp=bsc.

	Name	FWA
View	Institute of Public Health - Pontificia Universidad Javeriana	

2.2 * Upload the IRB/REC approval document(s):

Name	Modified Date	Owner
Certificado Comit_ etica -Diamond.pdf	8/24/2021 2:24 PM	Andres Ignacio Vecino Ortiz

3.0 * Is this research funded or have you applied for funding?
● **Yes** ○ No

3.1 * Is the sponsor a federal agency or another federal funding source?
○ Yes ● **No**

3.2 * Add your sponsor(s) (select one or more if applicable):

Name
Inter-American Development Bank

3.3 * If your sponsor's name does not appear in the list above, enter the name below:

Name

There are no items to display

3.4 Upload your Grant Contract / Agreement Title and complete the information requested:

Grant Contract / Agreement Title	Number	IP Number	Pending	Document
DIAMOND-R “Digital Applications to Monitor Novel coronavirus Disease and Response”	137799	Cynthia Polasko		

4.0 Will your study enroll patients or clients of the Maryland State Department of Health and Mental Hygiene (including those seeking care or assistance through county health departments), involve data compiled by or for DHMH, or be funded by or through the DHMH?

○ Yes ● **No**

Informed Consent for Adults

1.0 * Do you plan to obtain informed consent from your study participants?

(●) **Yes** ( ) No

1.1 * Which of these describes your informed consent process? Select all that apply.

- [ ] Consent document signed by participant
- [ ] Consent document and separate HIPAA authorization signed by participant
- [ ] Combined consent/HIPAA authorization document signed by participant
- [x] Oral consent script (e.g. participant will not sign the consent form)
- [ ] Oral consent script signed by witness not affiliated with study
- [ ] Consent obtained from adult's legally authorized representative (LAR)

1.2 Will you translate your English consent documents into another language?

(●) **Yes** ( ) No

1.2.1 Upload your Certificate of Translation for each language that attests to the qualifications of the translator. One certificate is adequate for multiple documents if the same person translated them.

Title	Modified Date	Owner
Certificate of Translation	9/14/2021 6:31 PM	Andres Ignacio Vecino Ortiz

1.3 * Provide consent documents for all languages.

	Title	Modified Date	Owner
View	Consent for qualitative interviews- English(0.01)	1/21/2022	Andres Ignacio Vecino Ortiz
View	Consent for qualitative interviews- Spanish(0.01)	1/21/2022	Andres Ignacio Vecino Ortiz
View	IVR Questionnaire (includes consent in both languages) (0.01)	1/21/2022	Andres Ignacio Vecino Ortiz

View: AP: Miscellaneous Documents

Miscellaneous Documents

1.0 Recruitment Material (Ads / Eligibility Screeners / Flyers / Listing Tools / Participant Correspondence during Recruitment and Data Collection / Results Letters / Scripts):

Title	Date Modified	Owner

There are no items to display

2.0 Surveys / Questionnaires / Guides / Research Instruments / General Correspondence:

	Title	Owner	Date Modified
View	IVR Questionnaire (in both languages)(0.01)	Andres Ignacio Vecino Ortiz	1/21/2022
View	Qualitative interviewing guide (Spanish version)(0.01)	Andres Ignacio Vecino Ortiz	1/20/2022
View	Qualitative interviewing guide(0.01)	Andres Ignacio Vecino Ortiz	1/20/2022

3.0 Medical Release Forms:

Title	Modified Date	Owner

There are no items to display

4.0 Other Documents:

Title	Modified Date	Owner
Letter of support from Ministry of Health	9/14/2021 6:32 PM	Andres Ignacio Vecino Ortiz

View: AP: Application End

Final Page

Before submitting this application to the IRB, you must invite all new study team personnel to participate on this study by clicking on the "Request Study Team Participation" activity from the menu.

1.0 Enter any additional information for the IRB Office:

Many thanks for reviewing this amendment. Upon conversation with Joan Petit, we were suggested to submit these changes as a public health surveillance determination form given the explicit requirement fo the Ministry of Health to pursue this work.

We did not include a tracked version because the form changed substantially.

2.0 With this submission, I affirm the following:

- I have read the protocol and this application.
- All questions on this application are answered truthfully and with appropriate completeness.
- Adequate resources and facilities are available to carry out the proposed research.
- Investigators and study team members will adhere to the current state and federal regulations, local law, international law, and institutional policy governing this research.
- I will ensure that all study personnel have the certification and/or credentialing required by law and by institutional policy to perform their assigned study activities, the appropriate training to conduct the portion of this study in which they are involved, and that they understand the study's standard operating procedures.
- I will ensure that all study team members (including students) have completed any and all required ethics, Good Clinical Practice, HIPAA and all other trainings needed for the study.
- I will ensure that the study personnel understand how to conduct the study in accordance with the terms of the JHSPH IRB approval and guidance.
- For studies using JHM clinical records: I certify that I have reviewed the JHM Privacy Office Data Protection Attestation and agree to be bound by its terms.

☑ *I affirm

3.0 If you are ready to submit this information to the IRB, answer "Yes" to the question below and click "Finish" at the top or bottom right of the page to send the application to the IRB. If you are not ready to submit this information to the IRB at this time, answer "No" to the question below, then click "Finish" and the information you have entered will be saved.

* Are you ready to submit this application to the IRB?

● **Yes** ○ No

La salud es de todos | Minsalud

Bogotá D.C., Colombia, September 9, 2020

Doctor
ANDRES VECINO
Assistant Scientist
International Health Department
Johns Hopkins Bloomberg School of Public Health

Subject: Request for support on developing a COVID-19 surveillance system using mobile phone technology.

Dear Dr. Vecino-Ortiz,

The Ministry of Health and Social Protection of Colombia promotes evidence-based decision-making for the Colombian health system. This is of special relevance in regards to the challenges that the country and the world face have faced amidst the Covid-19 pandemic.

Therefore, we want to express our interest in obtaining support to develop a COVID-19 surveillance system using mobile phone technology, which has been proposed in the framework of the project "DIAMOND-R Digital Applications to Monitor Novel coronavirus Disease and Response". The results of this project will be key to address the current response to the pandemic and also will help us to prepare for other current and future health challenges.

Regards

GERMAN ESCOBAR MORALES
Chief of staff to the Minister of Health and
Social Protection
Carrera 13 No. 32-76
Bogotá, D.C. Colombia

**IRB Use Only:** X Team ☐ IRB FC ☐
Review Type: Single Reviewer ☐ Group Discussion ☐ Convened ☐

Public Health Surveillance/Not Research Determination Form

Activities that meet the definition of "Public Health Surveillance" are not "human subjects research" and do not require ongoing IRB oversight. Whether or not they meet this definition, they must be conducted in accordance with institutional ethical standards. If you believe that your activity meets the definition, please answer the questions below and submit this form for consideration by the JHSPH IRB.

IRB Number	IRB00017868	
Study Title	Digital Applications to Monitor Novel coronavirus Disease and Response in Colombia - syndromic and vaccination surveillance	
Principal Investigator	Andres I. Vecino Ortiz	Email: avecino1@jhu.edu
Administrative Contact	Girlie Reyes	Email: girlie@jhu.edu

The "activity" referred to throughout the remainder of this document, and to which your responses/explanations pertain, refers to the surveillance, data collection, monitoring, evaluation "activity". It does not refer to the program, procedures, clinical services, or other action that is itself the target of the surveillance, data collection, monitoring, or evaluation. Please make clear this distinction in your responses.

1. Please provide a general description of the proposed activity and its objective(s):

The main objective of this activity is to conduct surveillance activities through interactive voice response (IVR) messages for COVID-19 in Colombia to support the broader surveillance effort of the Colombian government for both surveilling COVID-19 cases and adherence to vaccination.

In this work, we have been requested by the Government of Colombia to conduct this work focusing on three objectives: 1) syndromic surveillance for COVID-19; 2) surveillance on COVID-19 vaccine hesitancy and 3) test different messsaging strategies through IVR to provide information on COVID-19 vaccines.

1) We will deploy monthly syndromic surveys for COVID-19 using mobile phone technology (Mobile phone surveys or MPS) over a period of three to six months. The work will be conducted in one to five municipalities considered critical by this entity due to 1) low seroprevalence, 2) high infection rate, or 3) high proportion of vulnerable population. Preliminarily, the Ministry of Health has requested that we conduct the activities in the cities of Tunja, Manizales and Popayan. To this aim, we will obtain from the Colombian Ministry of Health an anonymized list of phone numbers registered at each of these municipalities from the national health insurance database (the database is called BDUA). The database will be provided by the Ministry of Health after signing a data confidentiality agreement with JHURA to safeguard the phone numbers of the potential respondents. These brief mobile phone surveys that lasts less than ten minutes and are deployed through Interactive Voice Response (IVR) or prerecorded calls, will ask questions about current respiratory symptoms, general health status,

COVID-19 vaccination and past known COVID-19 past infection. These surveys will allow the JHU team to detect changes in both the prevalence of respiratory symptoms and also vaccination trends, so we can report to the Ministry of Health to act quickly on COVID-19 outbreaks in these municipalities.

Using these initial surveillance calls, we will contact respondents that authorize being contacted again with two purposes:

1) A convenience sample of 10-20 individuals declaring not being vaccinated and who consent to be recontacted in the initial call will be called back to carry out a brief interview and ask them about their perceptions and social representations about COVID-19 vaccines. This effort will assist on the design of the messages that the Minsitry of Health has requested.

2) Individuals who have not been partially or fully vaccinated, and who consent to being recontacted will be called back and will be invited to participate in the messaging strategy. In this one, respondents who consent to participate will be randomized in two to three different arms and based on the qualitative interviews, they will be presented with different messages about COVID-19 vaccines. Individuals will receive messages and be followed up monthly for three months to assess the best mesaging strategy, so the government can deploy it to increase vaccination coverage.

2. To meet the definition of "public health surveillance", the activity must be "conducted by", "supported by", or "requested, ordered, required, or authorized by" a public health authority; this authority must have an official mandate with responsibility for public health matters.

Please describe the public authority and its relationship to the activity and those carrying out the activity:

This surveillance activity is conducted in support to the Colombian Ministry of Health. In particular, all monitoring activities are conducted in continued consultation with the Epidemiology Directorate of such Ministry.

3. The activity must be limited to that which is necessary to allow the public health authority to achieve its mandate to protect and maintain the health and welfare of the population for which it is responsible.

Please describe the mandate of the public health authority, and how the proposed work is limited to activities that allow the public authority to achieve its mandate:

The Colombian Ministry of Health has the constitutional mandate of preserving health of all residents in the national territory. This activity is focused on supporting the Ministry of Health by providing early signals of potential outbreaks of COVID-19, so the Ministry of Health can focus their activities in these municipalities. This is of particular relevance as country authorities are expecting a fourth Coronavirus wave associated to the Delta variant in Colombia.

4. Is the activity surveillance? While the Revised Common Rule does not explicitly define surveillance, the IRB will consider if the activity(ies): "monitor, assess, or investigate potential public health signals, onsets of disease outbreaks, or conditions of public health importance (including trends, signals, risk factors, patterns in diseases, or increases in injuries from using consumer products)."

Please describe the activity in sufficient detail such that the IRB can determine whether or not the activity can be considered public health surveillance:

This work constitutes surveillance as we will monitor 1) respiratory symptoms to detect patterns of increased risk for a COVID-19 outbreak 2) reasons for hesitancy and 3) types of messages that improve knowledge about COVID-19 vaccination.

1. We will deploy monthly surveys through Interactive Voice Response (IVR) in a random sample of phone numbers that are identified as belonging to individuals living in municipalities that are considered critical for syndromic surveillance. The criteria for critical locations will be done based on 1) vulnerable population (e.g. municipalities with a high percentage of senior population), 2) infection rate (e.g. municipalities with many active cases) and 3) seroprevalence (e.g. municipalities with low COVID-19 seroprevalence or vaccination rates). To focus the surveillance on specific municipalities, the Ministry of Health will provide us with the anonymized database of phone numbers registered in each of these municipalities through the BDUA (social health insurance registration) platform. Monthly surveys to individuals living in those municipalities will be deployed for three to six months to observe aggregated changes in the prevalence of respiratory symptoms.

The sample size for each municipality is calculated using the STEPS survey calculator for cluster random sampling, a recommended calculation for a repeated cross-sectional, population-based household surveys. The assumptions for the sample size are a 95% confidence (Z=1.96), margin of error = 0.05, Baseline prevalence of 0.5, yielding 385 complete surveys to be deployed each month per municipality. These sample sizes will change as parameters for sample size are calibrated after the first survey.

Trends in respiratory symptoms will be immediately reported to the Ministry of Health so they can follow up with their team of contact tracers and intensify active monitoring activities. Individuals reporting severe respiratory symptoms will also be immediately reported to the call center of the Ministry of Health for individual follow-up to that specific phone number.

All complete calls are expected to last a maximum of five minutes and will provide an incentive equivalent to around 1 USD.

2. A convenience sample of 10-20 individuals declaring being hesitant about the COVID-19 vaccines and that consent to be recontacted in the initial call will be called back to carry out a brief interview and ask them about their perceptions and social representations about COVID-19 vaccines. This effort will assist on the design of the COVID-19 messages requested by the government.

3) Individuals who have not been vaccinated (those who did not get any vaccine, did not get the second dose or did no get get the booster), and who consent to being recontacted will be called back and will be invited to participate in the messaging strategy. In this third objective, respondents who consent to participate will be randomized in two to three different arms (depending on the findings of the qualitative interviews) and will be presented with different messages about COVID-19 vaccines. Individuals will receive the different types of messages (two to three) and will be followed up monthly

for three months to assess whether they decided to get vaccinated.

Groups will be compared to assess the probability that a specific mesasaging strategy is signifcantly betetr than the other. With this aim, the sample size for each messaging group is N=200 which allows for the detection of a difference between groups of at least 10% with a double-tailed distribution at $p<0.05$.

5. Who is funding this activity, and for what period of time?

The Inter American Development Bank is funding this activity. This activity is included in a broader study for which further IRB submissions will be done and will las for 14 months under grant 137799, PI Vecino Ortiz, Coeus 21097978.

6. Please clarify the role of each partner/collaborator, and make clear the specific responsibilities of JHSPH personnel and subcontractors.

Our team is comprised by a combination of faculty from JHSPH DIH and the partner local university, Universidad Javeriana.

The project will be implemented through engagement of three teams: The JHU team, the Universidad Javeriana team and a steering committee with the Ministry of Health and the IADB.
The JHU team will lead the overall project and will lead and guide 1) data collection (sending surveys and messages to mobile phones); 2) data analysis; and 3) development of reports.

The Universidad Javeriana team will provide 1) the inputs for the development of the surveys and messages (recording of IVR surveys, development and translation of instruments, etc.), 2) the qualitative interviews 3) the messaging strategies 4) assistance and leadership on report development and 5) feedback on the regulatory processes for the deployment of the surveys. Local (Universidad Javeriana) IRB has already approved the entire proposal. Universidad Javeriana team will not have access to the mobile phone numbers provided by the Ministry of Health.

The steering committee will be comprised by the IADB and the Colombian Ministry of Health officials to obtain continuous feedback on the progress of the project and steer the direction of decisions that need to be taken.

Only JHU personnel will access anonymized information about the individuals being surveyed. Other teams will only have access to aggregated information.

7. For new data collection, comment on any ethical issues you foresee associated with recruitment, consent, privacy protections, and study procedures. What are the risks of this activity?

We will obtain anonymized data on current respiratory symptoms, general health status , vaccination status and hesitancy about COVID-19 vaccines from the respondents of the mobile phone numbers identified by the Ministry of Health as belonging to residents of the target municipalities. No attempt will be done to try to identify the identity of those receiving the surveys. In case a person reports severe respiratory symptoms, the phone number will be directed to the Ministry of Health contact center for follow up. Regarding recruitment, we will ensure that consent procedures pertaining to confidentiality and use of the data are locally appropriate and abide by local regulations. The local IRB of our partner university has already approved this protocol and copy of the IRB approval is attached. Regarding privacy protections and data storage risks, the data will be stored in a secured location at JHSPH servers in an anonymized format. Pertaining to procedures, the Ministry of Health will provide us the phone numbers under an agreement in which we can only use those phone numbers for the purposes of this epidemiological surveillance project. We will seek authorization from callers to call back to conduct follow up calls for syndromic surveys, qualitative interviewing, or vaccine messaging. Respondents from all phone numbers will be provided informed consent and if denied, they will never be called back. Phone numbers will be permanently deleted once the project is finished.

8. Please describe your data security protections. In particular, clarify what personal identifiers will be collected, retained, and/or removed. Make clear the risks, if any, associated with data collection, storage, sharing and general management.

The only potential identifier collected will be the phone number associated to the account. Neither names nor other identifiable data will be collected. Phone numbers will be retained during the data collection process to avoid calling back individuals who have not consented to be called for the duration of the surveillance activity will be requested. After the data collection process and analysis ends, all phone numbers will be deleted. A data agreement with the Minsitry of Health is being obtained.

9. What is the scale of this activity? How many individuals/households do you anticipate will be involved?

We will conduct 385 complete surveys, sampled from the universe of phone numbers provided by the Ministry of Health in the first month to each of the selected municipalities. In the following months, we are likely to change the sample size as sample calculation parameters change (e.g. prevalence of respiratory symptoms assumed at a conservative 50% for the first month). Conducting a maximum of 1,000 complete surveys per month for three to six months in a maximum of three locations, which yields a maximum of 18,000 complete surveys conducted through mobile phone users. Upon request of the Ministry of Health, we might extend the scope to other municipalities if budget allows.

For the qualitative work that will inform the design of the messages, 10-20 interviews are expected.

Fo the vaccine messaging strategy, we anticipate to have up to three different messaging groups, each with up to 200 individuals for a maximum of 600 individuals.

10. Will your data be stored or saved for future use? If yes, what kind of use do you anticipate, and by whom?<br>These data will be stored for future use in a complete anonymized format in which phone numbers will be removed.
11. Please describe your data sharing plan. Will the data be made publicly accessible, and if so, how? If by publication, will your partners/collaborators be co-authors?<br>We do not plan to share the raw data with partners, funders or the public. We might seek to publish this surveillance activity work for dissemination purposes in academic or noon-academic platforms. If that is the case, only aggregated data will be provided. Partners and collaborators will have the opportunity to participate as co-authors should they abide to ICJME rules.
Andres Ignacio Vecino Ortiz___<br>01/06/2022_<br>Signature of Principal Investigator Date

Notes	Module	English IVR	Spanish IVR	Response Options English	Response Options Spanish	Skipping Options & Other instructions English	Skipping Options & Other instructions Spanish
E_01	Error	I am sorry; the response you have entered is not one of the available options. I am going to repeat the question for you.	Discúlpenos; la respuesta que ingresó no es una de las opciones disponibles. Le repetiré la pregunta.			IF AN INVALID ANSWER IS SUBMITTED, PROMPT ERROR MESSAGE AND REPEAT QUESTION	SI SE ENVÍA UNA RESPUESTA INVÁLIDA, MENSAJE DE ERROR AND REPEAT QUESTION
E_02	Under 18	Sorry. Since you are under 18 years of age, you are not eligible for the survey. Thank you for your time.	Discúlpenos; como usted es menor de 18 años no podemos hacerle la encuesta. Gracias por su tiempo.				
E_03	No age	Sorry. Since we could not determine your age, we have to end the survey here. Thank you for your time.	Disculpe. Como no pudimos determinar su edad debemos terminar la encuesta ahora. Muchas gracias por su tiempo.				
I_1	Intro	Hello, this is a public health mobile phone survey led by Javeriana and Johns Hopkins Universities.<br><br>The objective is to know the vaccination status of Colombians against COVID-19.<br><br>For this, we have generated random mobile phone numbers that we are dialing. We will not ask for your name, identification data, or banking information. Your information will be anonymous and confidential according to the Data Protection Policy of the Universidad Javeriana, which you can consult on our web page.	Hola. Esta es una encuesta telefónica de salud pública liderada por las Universidades Javeriana y de Johns Hopkins.<br><br>El objetivo es conocer el estado de vacunación de los colombianos contra COVID-19.<br><br>Para esto hemos generado números celulares al azar a los que estamos marcando. No le preguntaremos su nombre, datos de identificación ni información bancaria. Su información será anónima y confidencial según la Política de Protección de Datos de la Universidad Javeriana que puede consultar en nuestra página web.	1 = YES<br>3 = NO<br><br>OTHER=E_01	1 = SÍ<br>3 = NO<br><br>OTRO= E_01	If the response is NO (3), skip to M_01 and END SURVEY<br><br>OTHER, READ E_01 and REPEAT THE QUESTION	Si la respuesta es NO (3), pase a M_01 y FINALIZA LA LLAMADA.<br>OTRO, LEER E_01 y REPETIR LA PREGUNTA.

		We will recharge you $4,000 if your cell phone is prepaid by completing the survey.<br><br>Following Law 1581 of 2012, do you authorize us to conduct this survey and process your data for public health research purposes?<br><br>For YES, press 1.<br>For NO, press 3.	Al completar la encuesta le haremos una recarga de $4.000 si su celular es prepago.<br><br>De acuerdo con la Ley 1581 de 2012 nos autoriza hacerle esta encuesta y el tratamiento de sus datos con fines de investigación en salud pública?<br><br>Para SI, presione 1.<br><br>Para NO, presione 3.				
Age	Demo	What is your age? Enter your age in years using your mobile phone's keypad. If you do not know your age, press 0.	¿Cuál es su edad? Ingrese su edad en años con el teclado de su teléfono móvil. Si no sabe su edad, presione 0.	RANGE= 18 –99<br>0= DO NOT KNOW<br>OTHER=E_01	RANGO = 18 –99<br>0 = NO SABE<br>OTRO = E_01	IF Range (18-99), GO TO Q_1_1<br><br>IF AGE <18yrs, READ E_02 and END SURVEY<br><br>IF OTHER, READ E_03 and END SURVEY<br><br>If 0, play M_01 and hang up.	Si Rango (18-99), ir a Q_1_1.<br><br>Si EDAD es <18 años, LEER E_02 y TERMINAR LA ENCUESTA.<br><br>Si es otra opción de las anteriores, LEER E_03 Y TERMINAR LA ENCUESTA.
Q_1_1	Vaccination	Have you been vaccinated against COVID-19? If yes, press 1. If no, press 3.	¿Ha sido usted vacunado contra COVID-19?<br><br>Sí se ha vacunado, presione 1;<br>si no se ha vacunado, presione 3.	1 = YES<br>3 = NO<br>OTHER=E_01	1 = SÍ<br>3 = NO<br>OTRO = E_01	IF 1, GO TO Q_2_1<br><br>IF 3, GO TO Q_1_2<br><br>OTHER, READ E_01 and REPEAT THE QUESTION	Si 1, ir a Q_2_1.<br><br>Si 3, ir a Q_1_2.<br><br>OTRO, LEER E_01 y REPETIR LA PREGUNTA.
Q_1_2	Vaccination	Would you like to receive the COVID-19 vaccine?	¿Usted quiere ponerse la vacuna contra COVID-19?<br><br>Sí quiere vacunarse, presione 1;	1= YES<br>3= NO	1= SI<br>3= NO	GO TO Q_1_3	ir a Q_1_3

		If you want to get vaccinated, press 1;<br>if you do not want to be vaccinated, press 3	si no quiere vacunarse, presione 3			OTHER, READ E_01 and REPEAT THE QUESTION	OTRO, LEER E_01 y REPETIR LA PREGUNTA.
Q_1_3	Vaccination	Why you have not been vaccinated? If there were no vaccines, press 1.<br><br>If you believe COVID vaccines don't work press 2.<br><br>If you believe COVID vaccines are not safe press 3.<br><br>If you believe you don't need the vaccine press 4.<br><br>If you have not vaccinated because you already had COVID press 5.<br><br>If you have not been able to get the vaccine press 6.<br><br>For other reasons, press 7.<br><br>If you don't know or don't want to respond, press 0,<br><br>Press 9 to repeat options	¿Por qué no se ha vacunado?<br><br>Porque no había vacunas presione 1.<br><br>Porque cree que las vacunas no funcionan presione 2.<br><br>Porque cree que las vacunas no son seguras presione 3.<br><br>Porque cree que no necesita la vacuna presione 4.<br><br>Porque ya le dio COVID presione 5.<br><br>Porque no ha podido ir presione 6.<br><br>Otras razones presione 7.<br><br>Si no sabe o no quiere responder presione 0.<br><br>Presione 9 para repetir las opciones	1= BECAUSE VACCINES WERE NOT AVAILABLE<br>2=BECAUSE I THINK THE VACCINE DOES NOT WORK<br>3=BECAUSE I THINK THE VACCINE IS NOT SAFE<br>4=BECAUSE I DON'T THINK I NEED IT<br>5=BECAUSE I ALREADY GOT COVID<br>6=BECAUSE I HAVE NOT BEEN ABLE TO GET THE VACCINE<br>7= OTHER REASONS<br><br>0= DON'T KNOW/ DON'T WANT TO RESPOND<br><br>9 = TO REPEAT OPTIONS<br>OTHER=E_01	1 = PORQUE NO HABÍA VACUNAS DISPONIBLES<br>2 = PORQUE CREO QUE LA VACUNA NO FUNCIONA<br><br>3=PORQUE CREO QUE LA VACUNA NO ES SEGURA<br><br>4= PORQUE CREO QUE NO LA NECESITO<br><br>5= PORQUE YA ME DIO COVID<br><br>6= PORQUE NO HE PODIDO IR A VACUNARME<br><br>7= OTRAS RAZONES<br><br>0= NO SABE/ NO DESEA RESPONDER<br><br>9 = PARA REPETIR LAS OPCIONES<br><br>OTHER=E_01	GO TO Q_3_1	Ir a Q_3_1
Q_2_1	Vaccination	How many doses of the Covid-19 have you received? If you had one dose, please press 1,<br>two doses, press 2.<br>three doses, press 3.<br>four doses, press 4.	¿Cuántas dosis de la vacuna ha recibido?<br>Presione 1 si se ha puesto una sola dosis.<br>Presione 2 si se ha puesto dos dosis.	1 = ONE DOSE<br>2 = TWO DOSES.<br>3 = THREE DOSES<br>4 = FOUR DOSES<br><br>0 = DON'T KNOW<br>OTHER=E_01	1 = UNA DOSIS<br>2 = DOS DOSIS.<br>3= TRES DOSIS<br>4= CUATRO DOSIS<br><br>0 = NO SABE<br>OTRO = E_01	GO TO Q_2_2<br><br>OTHER, READ E_01 AND REPEAT THE QUESTION	Ir a Q_2_2<br><br>OTRO, LEER E_01 y REPETIR LA PREGUNTA.

		If don't know, press 0	Presione 3 si se ha puesto tres dosis.<br>Presione 4 si ha recibido cuatro dosis.<br><br>Si no sabe, presione 0.				
Q_2_2	Vaccination	Which was the brand of the first vaccine you received?<br>For Coronavac of Sinovac, press 1. For Pfizer, press 2. For Astra Zeneca, press 3. For Moderna, press 4. For Janssen or Johnson and Johnson, press 5.<br>For other vaccine, press 6. If you don't know which vaccine you received, press 0.	¿Para la primera dosis, qué vacuna se puso?<br><br>Sinovac, presione 1.<br>Pfizer, presione 2.<br>Astra Zeneca, presione 3.<br>Moderna, presione 4.<br>Janssen o Johnson y Johnson, presione 5.<br>Otra vacuna, presione 6.<br><br>Si no sabe que vacuna recibió, presione 0.	1= Coronavac de Sinovac<br>2=Pfizer<br>3= Astra Zeneca<br>4= Moderna,<br>5=Janssen o Johnson y Johnson<br>6= Another vaccine<br>0= Don't know<br><br>OTHER=E_01	1= Coronavac de Sinovac<br>2=Pfizer<br>3= Astra Zeneca<br>4= Moderna<br>5= Janssen o Johnson y Johnson<br>6=Otra vacuna<br>0=No sabe qué vacuna recibió<br><br>OTRO=E_01	GO TO Q_2_3 IF Q_2_1>1<br><br>GO TO Q_3_1 IF Q_2_1<=1	IR A Q_2_3 SI Q_2_1>1<br><br>IR A Q_3_1 SI Q_2_1<=1
Q_2_3	Vaccination	Which was the brand of the second vaccine dose that-you received?<br>For Coronavac of Sinovac, press 1. For Pfizer, press 2. For Astra Zeneca, press 3. For Moderna, press 4. For Janssen or Johnson and Johnson, you would not need a second dose and therefore, please press 5. For other vaccine, press 6. If you don't know which vaccine or did not receive the second vaccine, press 0.	¿Para la segunda dosis, qué vacuna se puso?<br><br>Sinovac, presione 1.<br>Pfizer, presione 2.<br>Astra Zeneca, presione 3.<br>Moderna, presione 4.<br>Si fue Janssen o Johnson y Johnson y por tanto no se necesitaba segunda dosis, presione 5.-Otra vacuna, presione 6.<br><br>Si no sabe que vacuna recibió o no ha recibido la segunda vacuna, presione 0.	1= Coronavac de Sinovac<br>2=Pfizer<br>3=Astra Zeneca<br>4= Moderna,<br>5=Janssen o Johnson y Johnson<br>6= Another vaccine<br>0= Don't know/ has not received the second dose<br><br>OTHER=E_01	1= Coronavac de Sinovac<br>2=Pfizer<br>3= Astra Zeneca<br>4+ Moderna<br>5= Janssen o Johnson y Johnson<br>6=Otra vacuna<br>0=No sabe qué vacuna recibió/ no ha recibido la segunda dosis<br><br>OTRO=E_01	GO TO Q_2_4 IF Q_2_1>2<br><br>GO TO Q_3_1 IF Q_2_1<=2	IR A Q_2_4 SI Q_2_1>2<br><br>IR A Q_3_1 SI Q_2_1<=2
Q_2_4	Vaccination	Which was the brand of the third vaccine dose that you received?<br><br>For Coronavac of Sinovac, press 1. For Pfizer, press 2. For Astra Zeneca, press 3. For Moderna,	¿Para la tercera vacuna, qué vacuna se puso?<br><br>Sinovac, presione 1.<br>Pfizer, presione 2.<br>Astra Zeneca, presione 3.	1= Coronavac de Sinovac<br>2=Pfizer<br>3=Astra Zeneca<br>4= Moderna,	1= Coronavac de Sinovac<br>2=Pfizer<br>3= Astra Zeneca<br>4+ Moderna	GO TO Q_2_5 IF Q_2_1>3<br><br>GO TO Q_3_1 IF Q_2_1<=3	IR A Q_2_5 SI Q_2_1>3<br><br>IR A Q_3_1 SI Q_2_1<=3

		press 4. For Janssen or Johnson and Johnson, please press 5. For other vaccine, press 6. If you don't know which vaccine or did not receive the second vaccine, press 0.	Moderna, presione 4.<br>Si fue Janssen o Johnson y Johnson, presione 5. -Otra vacuna, presione 6.<br><br>Si no sabe qué vacuna recibió o no ha recibido el primer refuerzo, presione 0.	5=Janssen o Johnson y Johnson<br>6= Another vaccine<br>0= Don't know/ has not received the second dose<br><br>OTHER=E_01	5= Janssen o Johnson y Johnson<br>6=Otra vacuna<br>0=No sabe qué vacuna recibió/ no ha recibido la segunda dosis<br><br>OTRO=E_01		
Q_2_5	Vaccination	Which was the brand of the fourth vaccine dose that you received?<br><br>For Coronavac of Sinovac, press 1. For Pfizer, press 2. For Astra Zeneca, press 3. For Moderna, press 4. For Janssen or Johnson and Johnson, please press 5. For other vaccine, press 6. If you don't know which vaccine or did not receive the second vaccine, press 0.	¿Para la cuarta vacuna, qué vacuna se puso?<br><br>Sinovac, presione 1.<br>Pfizer, presione 2.<br>Astra Zeneca, presione 3.<br>Moderna, presione 4.<br>Si fue Janssen o Johnson y Johnson, presione 5.-Otra vacuna, presione 6.<br><br>Si no sabe qué vacuna recibió o no ha recibido el segundo refuerzo, presione 0.	1= Coronavac de Sinovac<br>2=Pfizer<br>3=Astra Zeneca<br>4= Moderna,<br>5=Janssen o Johnson y Johnson<br>6= Another vaccine<br>0= Don't know/ has not received the second dose<br><br>OTHER=E_01	1= Coronavac de Sinovac<br>2=Pfizer<br>3= Astra Zeneca<br>4= Moderna<br>5= Janssen o Johnson y Johnson<br>6=Otra vacuna<br>0=No sabe qué vacuna recibió/ no ha recibido la segunda dosis<br><br>OTRO=E_01	GO TO Q_3_1	Ir a Q_3_1
Q_3_1	Ever prevalence	Thank you for answering these questions so far. Now we have a question for you regarding previous COVID-19 infection and vaccination. Have you ever had COVID-19? If you had COVID-19 confirmed by healthcare personnel, press 1;<br>if you believe you had COVID-19 but it was never confirmed, press 3. If you don't have ever had COVID-19, press 5.	¿Ha tenido usted COVID-19?<br><br>Si ha tenido COVID-19 confirmado por personal de salud, presione 1;<br>Si usted cree que ha tenido COVID-19, pero nunca fue confirmado, presione 3.<br>Si usted cree que nunca ha tenido COVID-19, presione 5.	1 = YES, CONFIRMED<br>3= YES, SUSPECTED<br>5 = NO<br>OTHER=E_01	1 = SÍ, CONFIRMADO<br>3 = SÍ, SOSPECHA<br>5= NO<br>OTRO = E_01	GO TO Q_03_02<br><br>OTHER, READ E_01 and REPEAT THE QUESTION	Ir a Q_03_02<br><br>OTRO, LEER E_01 y REPETIR LA PREGUNTA.

Q_03_02	Demo	What is your gender? If you are a man, press 1. If you are a woman, then press 3. You may press 5 in case of other gender.	¿Cuál es su género?<br>Para hombre, presione 1.<br>Para mujer, presione 3.<br>Si se reconoce en otro género presione 5.	1 = MAN<br>3 = WOMAN<br>5 = OTHER GENDER<br>OTHER=E_01	1 = HOMBRE<br>3 = MUJER<br>5 = OTRO GÉNERO<br>OTRO = E_01	GO TO Q_03_3<br><br>OTHER, READ E_01 and REPEAT THE QUESTION	Ir a Q_03_3<br><br>OTRO, LEE E_01 y REPETIR LA PREGUNTA.
Q_03_3	Demo	Do you live in an urban area (within City Corporation or municipality area), or in a rural area? If you live in an urban area, press 1. If you live in a rural area, press 3.	¿Vive en un área urbana o rural?<br>Por ejemplo:<br>- Área urbana es la cabecera municipal.<br>- Área rural es una vereda, un corregimiento o rural disperso.<br><br>Si vive en un área urbana, pulse 1.<br>Si vive en una área rural, pulse 3.	1 = Urban area<br>3 = Rural area	1 = Área urbana<br>3 = Área rural	GO TO Q_03_4<br><br>OTHER, READ E_01 and REPEAT THE QUESTION	Ir a Q_03_4<br><br>OTRO, LEE E_01 y REPETIR LA PREGUNTA.
Q_03_4	Demo	What is your highest education level? If you don't have any studies press 1, if your highest educational level is elementary school press 2, if it is high school press 3, if it is a technical degree press 4, if you have an undergraduate or graduate degree press 5	¿Cuál es el nivel educativo más alto alcanzado por usted?<br>Si usted no ha cursado ningún grado presione 1;<br>Si cursó algún grado de primaria presione 2;<br>Si cursó algún grado de secundaria o bachillerato presione 3;<br>Si cursó un grado técnico o tecnológico presione 4;<br>Si usted estudió un grado universitario presione 5.	1= I don't have any studies<br><br>2= My highest educational level is elementary school<br><br>3= My highest educational level is middle or high school<br><br>4= I have a technical or technological degree<br><br>5= I have an undergraduate or graduate degree	1 = Ninguno<br>2 = Primaria<br>3 = Secundaria<br>4 = Técnico o Tecnológico<br>5 = Universitario o posgrado	GO TO Q_04<br><br>OTHER, READ E_01 and REPEAT THE QUESTION	Ir a Q_04<br><br>OTRO, LEE E_01 y REPETIR LA PREGUNTA.
ELIGIBILITY FOR MESSAGING	NO AUDIO	NO AUDIO	NO AUDIO	NO AUDIO	NO AUDIO	CONTINUE WITH MESSAGE:<br><br>If Q_2_1<2<br><br>If Q_2_1=2 AND Q_2_2=5 AND Age>50	CONTINUAR CON MENSAJE:<br><br>Si Q_2_1<2<br><br>Si Q_2_1=2 Y Q_2_2=5 Y Age>50

						If Q_2_1=3 AND Q_2_2!=5 AND Age>50<br><br>GO TO Q_13<br><br>If Q_2_1=2 AND Q_2_2!=5 AND Age<50<br><br>If Q_2_1=3 AND Q_2_2!=5 AND Age<50<br><br>If Q_2_1=3 AND Q_2_2=5 AND Age>50<br><br>If Q_2_1 = 4	Si Q_2_1=3 Y Q_2_2!=5 Y Age>50<br><br>IR A Q_13<br><br>Si Q_2_1=2 Y Q_2_2!=5 Y Age<50<br><br>SI Q_2_1=3 Y Q_2_2!=5 Y Age<50<br><br>Si Q_2_1=3 Y Q_2_2=5 Y Age>50<br><br>Si Q_2_1 = 4
Q_04	Intro Message	Your answers have been very helpful and thanks to them we have chosen you to participate in a short national program that aims to know the vaccination status of Colombians against COVID-19 during the next two months. Every time we call you, we will recharge your mobile phone with 4,000 pesos if it is prepaid.<br><br>If you accept, we will make only two calls per week.<br><br>If you would like to participate in this program, please press 1,<br>If you do not wish to participate, please press 3.	Sus respuestas han sido muy valiosas y gracias a estas le hemos elegido para participar<br>en un corto programa nacional que tiene como objetivo conocer el estado de vacunación de los colombianos contra COVID-19durante los próximos dos meses. Cada vez que le llamemos le haremos una recarga de 4,000 pesos en su teléfono celular si es prepago.<br><br>Si usted acepta, le haremos solo dos llamadas por semana.<br><br>Si le gustaría participar de este programa, presione 1,<br>Si no desea participar, por favor presione 3.	1 = YES<br>3 = NO<br>OTHER=E_01	1 = SÍ<br>3 = NO<br>OTRO = E_01	IF 1, GO TO Q_13<br><br>IF 3, GO TO Q_13 and end the call (use the M_02 audio for ending)	Si 1, ir a Q_13<br><br>Si 3, ir a Q_13

Q_13	Exit	Is your phone number prepaid or postpaid?<br>For prepaid, press 1.<br>For postpaid, press 3.	¿Su número telefónico es prepago o postpago?<br>Para prepago, presione 1.<br>Para postpago, presione 3.	1 = PREPAID<br>3 = POSTPAID<br>OTHER=E_01	1 = PREPAGO<br>3 = POSTPAGO<br>OTRO = E_01	IF 1, GO TO M_01<br><br>IF 3, GO TO M_03<br><br>OTHER, READ E_01 and REPEAT THE QUESTION	Si 1, ir a M_01.<br><br>Si 3, ir a M_03<br><br>OTRO, LEER E_01 y REPETIR LA PREGUNTA.
M_01	Thank You	You have given us very valuable information and we are sincerely grateful. We will credit your account if it is prepaid with 4,000 pesos, and will call you again soon	Usted nos ha provisto una información muy valiosa y estamos muy agradecidos. Le recargaremos su teléfono móvil, si es prepago, con 4,000 pesos y lo llamaremos de nuevo pronto.				
M_02	Thank You	The survey has ended. Universidad Javeriana and Johns Hopkins thank you for responding the call. Remember to get the vaccine, wash your hands and use masks.	¡Muchas gracias, hemos terminado la encuesta! Las Universidades Javeriana y de Johns Hopkins le agradecemos por atender esta llamada. Recuerde vacunarse, lavarse las manos cada vez que pueda y usar su tapabocas.				
M_03	Thank You	You have given us very valuable information and we are sincerely grateful. We will call you again soon	Usted nos ha provisto una información muy valiosa y estamos muy agradecidos. Le llamaremos de nuevo pronto.				
M_04	Reconnect message	Hi, this is the Pontificia Universidad Javeriana de Bogota, and Johns Hopkins University calling you back. It seems that the call dropped. We'll continue the COVID-19 survey where you left off.<br><br>If you would like to continue with the survey, press 1. If you do not want to continue with the call, press 3.	Hola. La Pontificia Universidad Javeriana de Bogotá y la Universidad de Johns Hopkins le esta regresado la llamada para continuar con la encuesta sobre COVID-19 que usted estaba realizando. Al parecer la llamada se cayó.<br><br>Si desea continuar con la encuesta, presione 1. Si no desea continuar con la llamada, presione 3.	1: Continue with the survey.<br><br>3: Do not continue with the survey.	1: Continuar con la encuesta.<br><br>3: No continuar con la encuesta.	IF 1, CONTINUE THE SURVEY WHERE THEY LEFT OFF.<br><br>IF 3, GO TO M_01	SI 1, CONTINUAR LA ENCUESTA DONDE LA DEJARON.<br><br>Si 3, ir a M_01

Subsequent calls

Intro Message _2		Hi, this is again the Javeriana University and Johns Hopkins Vaccination Survey for COVID-19.<br><br>At the end of the call, which will take less than five minutes, we will give you a 4,000 peso recharge on your prepaid cell phone. We will ask you a question about vaccination for COVID-19.	Hola. Esta es de nuevo la encuesta de la Universidad Javeriana y Johns Hopkins sobre vacunación para COVID-19.<br><br>Al finalizar la llamada, que tomará menos de cinco minutos, le<br>ha<br>remos una recarga de 4,000 pesos en su teléfono celular prepago. Le	1 = YES<br>3 = NO<br>OTHER=E_01	1 = SÍ<br>3 = NO<br>OTRO = E_01	IF 1, GO TO VACCINE<br><br>IF 3, GO TO M_02	Si 1, Ir a Vaccine<br><br>SI 3, ir a M_02

		Do you agree to continue?<br><br>If yes, press 1,<br>If you do not accept, press 3.	haremos una pregunta sobre vacunación para COVID-19.<br><br>¿Acepta continuar?<br><br>Si acepta, presione 1,<br>Si no acepta presione 3.				
Vaccine		Have you been vaccinated against COVID-19 in the last week? If YES, press 1. If NO, press 3	Se ha usted vacunado contra COVID-19 en la última semana? Si se ha vacunado contra COVID-19 en la última semana, presione 1. Si no se ha vacunado en la última semana, presione 3.	1 = YES<br>3 = NO<br>OTHER=E_01	1 = SÍ<br>3 = NO<br>OTRO = E_01	IF 1, GO TO Pre_postpaid<br><br>IF 3, GO TO Pre_postpaid	Si 1, Ir a M_01<br>SI 3, Ir a Pre_postpaid
Pre_post paid	Pre or postpaid	Finally, we would like to confirm if your phone line is prepaid or postpaid.<br><br>For prepaid dial 1.<br>For postpaid dial 3.	Para finalizar, nos gustaría confirmar si su línea de teléfono es prepago o pospago.<br><br>Para prepago marque 1.<br>Para postpago marque 3.	1 = prepaid<br>3 = postpaid<br>OTHER=E_01	1 = prepago<br>3 = postpaid<br>OTRO = E_01	IF 1, GO TO M_01<br><br>IF 3, GO TO M_03	Si 1, Ir a M_01<br>SI 3, Ir a M_03
M_01	Thank You	You have given us very valuable information and we are sincerely grateful. We will credit your account if it is prepaid with 4,000 pesos, and will call you again soon	Usted nos ha provisto una información muy valiosa y estamos muy agradecidos. Le recargaremos su teléfono móvil, si es prepago, con 4,000 pesos y lo llamaremos de nuevo pronto.				
M_02	Thank You	The survey has ended. Universidad Javeriana and Johns Hopkins thank you for responding the call. Remember to get the vaccine, wash your hands and use masks.	¡Muchas gracias, hemos terminado la encuesta! Las Universidades Javeriana y de Johns Hopkins le agradecemos por atender esta llamada. Recuerde vacunarse, lavarse las manos cada vez que pueda y usar su tapabocas.				
M_03	Thank You	You have given us very valuable information and we are sincerely grateful. We will call you again soon	Usted nos ha provisto una información muy valiosa y estamos muy agradecidos. Le llamaremos de nuevo pronto.				

M_04	Reconnect message	Hi, this is the Pontificia Universidad Javeriana de Bogota, and Johns Hopkins University calling you back. It seems that the call dropped. We'll continue the COVID-19 survey where you left off.<br><br>If you would like to continue with the survey, press 1. If you do not want to continue with the call, press 3.	Hola. La Pontificia Universidad Javeriana de Bogotá y la Universidad de Johns Hopkins le esta regresado la llamada para continuar con la encuesta sobre COVID-19 que usted estaba realizando. Al parecer la llamada se cayó.<br><br>Si desea continuar con la encuesta, presione 1. Si no desea continuar con la llamada, presione 3.	1: Continue with the survey.<br><br>3: Do not continue with the survey.	1: Continuar con la encuesta.<br><br>3: No continuar con la encuesta.	IF 1, CONTINUE THE SURVEY WHERE THEY LEFT OFF.<br><br>IF 3, GO TO M_01	SI 1, CONTINUAR LA ENCUESTA DONDE LA DEJARON.<br><br>Si 3, ir a M_01

Notes	Module	English IVR	Spanish IVR	Response Options English	Response Options Spanish	Skipping Options & Other instructions English	Skipping Options & Other instructions Spanish
E_01	Error	I am sorry; the response you have entered is not one of the available options. I am going to repeat the question for you.	Discúlpenos; la respuesta que ingresó no es una de las opciones disponibles. Le repetiré la pregunta.			IF AN INVALID ANSWER IS SUBMITTED, PROMPT ERROR MESSAGE AND REPEAT QUESTION	SI SE ENVÍA UNA RESPUESTA INVÁLIDA, MENSAJE DE ERROR AND REPEAT QUESTION
E_02	Under 18	Sorry. Since you are under 18 years of age, you are not eligible for the survey. Thank you for your time.	Discúlpenos; como usted es menor de 18 años no podemos hacerle la encuesta. Gracias por su tiempo.				
E_03	No age	Sorry. Since we could not determine your age, we have to end the survey here. Thank you for your time.	Disculpe. Como no pudimos determinar su edad debemos terminar la encuesta ahora. Muchas gracias por su tiempo.				
I_1	Intro	Hello, this is a public health mobile phone survey led by Javeriana and Johns Hopkins Universities.<br><br>The objective is to know the vaccination status of Colombians against COVID-19.<br><br>For this, we have generated random mobile phone numbers that we are dialing. We will not ask for your name, identification data, or banking information. Your information will be anonymous and confidential according to the Data Protection Policy of the Universidad Javeriana, which you can consult on our web page.	Hola. Esta es una encuesta telefónica de salud pública liderada por las Universidades Javeriana y de Johns Hopkins.<br><br>El objetivo es conocer el estado de vacunación de los colombianos contra COVID-19.<br><br>Para esto hemos generado números celulares al azar a los que estamos marcando. No le preguntaremos su nombre, datos de identificación ni información bancaria. Su información será anónima y confidencial según la Política de Protección de Datos de la Universidad Javeriana que puede consultar en nuestra página web.	1 = YES<br>3 = NO<br><br>OTHER=E_01	1 = SÍ<br>3 = NO<br><br>OTRO= E_01	If the response is NO (3), skip to M_01 and END SURVEY<br><br>OTHER, READ E_01 and REPEAT THE QUESTION	Si la respuesta es NO (3), pase a M_01 y FINALIZA LA LLAMADA.<br>OTRO, LEER E_01 y REPETIR LA PREGUNTA.

		We will recharge you $4,000 if your cell phone is prepaid by completing the survey.<br><br>Following Law 1581 of 2012, do you authorize us to conduct this survey and process your data for public health research purposes?<br><br>For YES, press 1.<br>For NO, press 3.	Al completar la encuesta le haremos una recarga de $4.000 si su celular es prepago.<br><br>De acuerdo con la Ley 1581 de 2012 nos autoriza hacerle esta encuesta y el tratamiento de sus datos con fines de investigación en salud pública?<br><br>Para SI, presione 1.<br><br>Para NO, presione 3.				
Age	Demo	What is your age? Enter your age in years using your mobile phone's keypad. If you do not know your age, press 0.	¿Cuál es su edad? Ingrese su edad en años con el teclado de su teléfono móvil. Si no sabe su edad, presione 0.	RANGE= 18 –99<br>0= DO NOT KNOW<br>OTHER=E_01	RANGO = 18 –99<br>0 = NO SABE<br>OTRO = E_01	IF Range (18-99), GO TO Q_1_1<br><br>IF AGE <18yrs, READ E_02 and END SURVEY<br><br>IF OTHER, READ E_03 and END SURVEY<br><br>If 0, play M_01 and hang up.	Si Rango (18-99), ir a Q_1_1.<br><br>Si EDAD es <18 años, LEER E_02 y TERMINAR LA ENCUESTA.<br><br>Si es otra opción de las anteriores, LEER E_03 Y TERMINAR LA ENCUESTA.
Q_1_1	Vaccination	Have you been vaccinated against COVID-19? If yes, press 1. If no, press 3.	¿Ha sido usted vacunado contra COVID-19?<br><br>Sí se ha vacunado, presione 1;<br>si no se ha vacunado, presione 3.	1 = YES<br>3 = NO<br>OTHER=E_01	1 = SÍ<br>3 = NO<br>OTRO = E_01	IF 1, GO TO Q_2_1<br><br>IF 3, GO TO Q_1_2<br><br>OTHER, READ E_01 and REPEAT THE QUESTION	Si 1, ir a Q_2_1.<br><br>Si 3, ir a Q_1_2.<br><br>OTRO, LEER E_01 y REPETIR LA PREGUNTA.
Q_1_2	Vaccination	Would you like to receive the COVID-19 vaccine?	¿Usted quiere ponerse la vacuna contra COVID-19?<br><br>Sí quiere vacunarse, presione 1;	1= YES<br>3= NO	1= SI<br>3= NO	GO TO Q_1_3	ir a Q_1_3

		If you want to get vaccinated, press 1;<br>if you do not want to be vaccinated, press 3	si no quiere vacunarse, presione 3			OTHER, READ E_01 and REPEAT THE QUESTION	OTRO, LEER E_01 y REPETIR LA PREGUNTA.
Q_1_3	Vaccination	Why you have not been vaccinated? If there were no vaccines, press 1.<br><br>If you believe COVID vaccines don't work press 2.<br><br>If you believe COVID vaccines are not safe press 3.<br><br>If you believe you don't need the vaccine press 4.<br><br>If you have not vaccinated because you already had COVID press 5.<br><br>If you have not been able to get the vaccine press 6.<br><br>For other reasons, press 7.<br><br>If you don't know or don't want to respond, press 0,<br><br>Press 9 to repeat options	¿Por qué no se ha vacunado?<br><br>Porque no había vacunas presione 1.<br><br>Porque cree que las vacunas no funcionan presione 2.<br><br>Porque cree que las vacunas no son seguras presione 3.<br><br>Porque cree que no necesita la vacuna presione 4.<br><br>Porque ya le dio COVID presione 5.<br><br>Porque no ha podido ir presione 6.<br><br>Otras razones presione 7.<br><br>Si no sabe o no quiere responder presione 0.<br><br>Presione 9 para repetir las opciones	1= BECAUSE VACCINES WERE NOT AVAILABLE<br>2=BECAUSE I THINK THE VACCINE DOES NOT WORK<br>3=BECAUSE I THINK THE VACCINE IS NOT SAFE<br>4=BECAUSE I DON'T THINK I NEED IT<br>5=BECAUSE I ALREADY GOT COVID<br>6=BECAUSE I HAVE NOT BEEN ABLE TO GET THE VACCINE<br>7= OTHER REASONS<br><br>0= DON'T KNOW/ DON'T WANT TO RESPOND<br><br>9 = TO REPEAT OPTIONS<br>OTHER=E_01	1 = PORQUE NO HABÍA VACUNAS DISPONIBLES<br>2 = PORQUE CREO QUE LA VACUNA NO FUNCIONA<br><br>3=PORQUE CREO QUE LA VACUNA NO ES SEGURA<br><br>4= PORQUE CREO QUE NO LA NECESITO<br><br>5= PORQUE YA ME DIO COVID<br><br>6= PORQUE NO HE PODIDO IR A VACUNARME<br><br>7= OTRAS RAZONES<br><br>0= NO SABE/ NO DESEA RESPONDER<br><br>9 = PARA REPETIR LAS OPCIONES<br><br>OTHER=E_01	GO TO Q_3_1	Ir a Q_3_1
Q_2_1	Vaccination	How many doses of the Covid-19 have you received? If you had one dose, please press 1,<br>two doses, press 2.<br>three doses, press 3.<br>four doses, press 4.	¿Cuántas dosis de la vacuna ha recibido?<br>Presione 1 si se ha puesto una sola dosis.<br>Presione 2 si se ha puesto dos dosis.	1 = ONE DOSE<br>2 = TWO DOSES.<br>3 = THREE DOSES<br>4 = FOUR DOSES<br><br>0 = DON'T KNOW<br>OTHER=E_01	1 = UNA DOSIS<br>2 = DOS DOSIS.<br>3= TRES DOSIS<br>4= CUATRO DOSIS<br><br>0 = NO SABE<br>OTRO = E_01	GO TO Q_2_2<br><br>OTHER, READ E_01 AND REPEAT THE QUESTION	Ir a Q_2_2<br><br>OTRO, LEER E_01 y REPETIR LA PREGUNTA.

		If don’t know, press 0	Presione 3 si se ha puesto tres dosis.<br>Presione 4 si ha recibido cuatro dosis.<br><br>Si no sabe, presione 0.				
Q_2_2	Vaccination	Which was the brand of the first vaccine you received?<br>For Coronavac of Sinovac, press 1. For Pfizer, press 2. For Astra Zeneca, press 3. For Moderna, press 4. For Janssen or Johnson and Johnson, press 5.<br>For other vaccine, press 6. If you don’t know which vaccine you received, press 0.	¿Para la primera dosis, qué vacuna se puso?<br><br>Sinovac, presione 1.<br>Pfizer, presione 2.<br>Astra Zeneca, presione 3.<br>Moderna, presione 4.<br>Janssen o Johnson y Johnson, presione 5.<br>Otra vacuna, presione 6.<br><br>Si no sabe que vacuna recibió, presione 0.	1= Coronavac de Sinovac<br>2=Pfizer<br>3= Astra Zeneca<br>4= Moderna,<br>5=Janssen o Johnson y Johnson<br>6= Another vaccine<br>0= Don’t know<br><br>OTHER=E_01	1= Coronavac de Sinovac<br>2=Pfizer<br>3= Astra Zeneca<br>4= Moderna<br>5= Janssen o Johnson y Johnson<br>6=Otra vacuna<br>0=No sabe qué vacuna recibió<br><br>OTRO=E_01	GO TO Q_2_3 IF Q_2_1>1<br><br>GO TO Q_3_1 IF Q_2_1<=1	IR A Q_2_3 SI Q_2_1>1<br><br>IR A Q_3_1 SI Q_2_1<=1
Q_2_3	Vaccination	Which was the brand of the second vaccine dose that-you received?<br>For Coronavac of Sinovac, press 1. For Pfizer, press 2. For Astra Zeneca, press 3. For Moderna, press 4. For Janssen or Johnson and Johnson, you would not need a second dose and therefore, please press 5. For other vaccine, press 6. If you don’t know which vaccine or did not receive the second vaccine, press 0.	¿Para la segunda dosis, qué vacuna se puso?<br><br>Sinovac, presione 1.<br>Pfizer, presione 2.<br>Astra Zeneca, presione 3.<br>Moderna, presione 4.<br>Si fue Janssen o Johnson y Johnson y por tanto no se necesitaba segunda dosis, presione 5.-Otra vacuna, presione 6.<br><br>Si no sabe que vacuna recibió o no ha recibido la segunda vacuna, presione 0.	1= Coronavac de Sinovac<br>2=Pfizer<br>3=Astra Zeneca<br>4= Moderna,<br>5=Janssen o Johnson y Johnson<br>6= Another vaccine<br>0= Don’t know/ has not received the second dose<br><br>OTHER=E_01	1= Coronavac de Sinovac<br>2=Pfizer<br>3= Astra Zeneca<br>4+ Moderna<br>5= Janssen o Johnson y Johnson<br>6=Otra vacuna<br>0=No sabe qué vacuna recibió/ no ha recibido la segunda dosis<br><br>OTRO=E_01	GO TO Q_2_4 IF Q_2_1>2<br><br>GO TO Q_3_1 IF Q_2_1<=2	IR A Q_2_4 SI Q_2_1>2<br><br>IR A Q_3_1 SI Q_2_1<=2
Q_2_4	Vaccination	Which was the brand of the third vaccine dose that you received?<br>For Coronavac of Sinovac, press 1. For Pfizer, press 2. For Astra Zeneca, press 3. For Moderna, press 4. For Janssen or Johnson	¿Para la tercera vacuna, qué vacuna se puso?<br><br>Sinovac, presione 1.<br>Pfizer, presione 2.<br>Astra Zeneca, presione 3.	1= Coronavac de Sinovac<br>2=Pfizer<br>3=Astra Zeneca<br>4= Moderna,	1= Coronavac de Sinovac<br>2=Pfizer<br>3= Astra Zeneca<br>4= Moderna	GO TO Q_2_5 IF Q_2_1>3<br><br>GO TO Q_3_1 IF Q_2_1<=3	IR A Q_2_5 SI Q_2_1>3<br><br>IR A Q_3_1 SI Q_2_1<=3

		and Johnson, please press 5. For other vaccine, press 6. If you don’t know which vaccine or did not receive the second vaccine, press 0.	Moderna, presione 4.<br>Si fue Janssen o Johnson y Johnson, presione 5. -Otra vacuna, presione 6.<br><br>Si no sabe qué vacuna recibió o no ha recibido el primer refuerzo, presione 0.	5=Janssen o Johnson y Johnson<br>6= Another vaccine<br>0= Don’t know/ has not received the second dose<br><br>OTHER=E_01	5= Janssen o Johnson y Johnson<br>6=Otra vacuna<br>0=No sabe qué vacuna recibió/ no ha recibido la segunda dosis<br><br>OTRO=E_01		
Q_2_5	Vaccination	Which was the brand of the fourth vaccine dose that you received?<br><br>For Coronavac of Sinovac, press 1. For Pfizer, press 2. For Astra Zeneca, press 3. For Moderna, press 4. For Janssen or Johnson and Johnson, please press 5. For other vaccine, press 6. If you don’t know which vaccine or did not receive the second vaccine, press 0.	¿Para la cuarta vacuna, qué vacuna se puso?<br><br>Sinovac, presione 1.<br>Pfizer, presione 2.<br>Astra Zeneca, presione 3.<br>Moderna, presione 4.<br>Si fue Janssen o Johnson y Johnson, presione 5.-Otra vacuna, presione 6.<br><br>Si no sabe qué vacuna recibió o no ha recibido el segundo refuerzo, presione 0.	1= Coronavac de Sinovac<br>2=Pfizer<br>3=Astra Zeneca<br>4= Moderna,<br>5=Janssen o Johnson y Johnson<br>6= Another vaccine<br>0= Don’t know/ has not received the second dose<br><br>OTHER=E_01	1= Coronavac de Sinovac<br>2=Pfizer<br>3= Astra Zeneca<br>4= Moderna<br>5= Janssen o Johnson y Johnson<br>6=Otra vacuna<br>0=No sabe qué vacuna recibió/ no ha recibido la segunda dosis<br><br>OTRO=E_01	GO TO Q_3_1	Ir a Q_3_1
Q_3_1	Ever prevalence	Thank you for answering these questions so far. Now we have a question for you regarding previous COVID-19 infection and vaccination. Have you ever had COVID-19? If you had COVID-19 confirmed by healthcare personnel, press 1;<br>if you believe you had COVID-19 but it was never confirmed, press 3. If you don’t have ever had COVID-19, press 5.	¿Ha tenido usted COVID-19?<br><br>Si ha tenido COVID-19 confirmado por personal de salud, presione 1;<br>Si usted cree que ha tenido COVID-19, pero nunca fue confirmado, presione 3.<br>Si usted cree que nunca ha tenido COVID-19, presione 5.	1 = YES, CONFIRMED<br>3= YES, SUSPECTED<br>5 = NO<br>OTHER=E_01	1 = SÍ, CONFIRMADO<br>3 = SÍ, SOSPECHA<br>5= NO<br>OTRO = E_01	GO TO Q_03_02<br><br>OTHER, READ E_01 and REPEAT THE QUESTION	Ir a Q_03_02<br><br>OTRO, LEER E_01 y REPETIR LA PREGUNTA.

Q_03_02	Demo	What is your gender? If you are a man, press 1. If you are a woman, then press 3. You may press 5 in case of other gender.	¿Cuál es su género?<br>Para hombre, presione 1.<br>Para mujer, presione 3.<br>Si se reconoce en otro género presione 5.	1 = MAN<br>3 = WOMAN<br>5 = OTHER GENDER<br>OTHER=E_01	1 = HOMBRE<br>3 = MUJER<br>5 = OTRO GÉNERO<br>OTRO = E_01	GO TO Q_03_3<br><br>OTHER, READ E_01 and REPEAT THE QUESTION	Ir a Q_03_3<br><br>OTRO, LEE E_01 y REPETIR LA PREGUNTA.
Q_03_3	Demo	Do you live in an urban area (within City Corporation or municipality area), or in a rural area? If you live in an urban area, press 1. If you live in a rural area, press 3.	¿Vive en un área urbana o rural?<br>Por ejemplo:<br>- Área urbana es la cabecera municipal.<br>- Área rural es una vereda, un corregimiento o rural disperso.<br><br>Si vive en un área urbana, pulse 1.<br>Si vive en una área rural, pulse 3.	1 = Urban area<br>3 = Rural area	1 = Área urbana<br>3 = Área rural	GO TO Q_03_4<br><br>OTHER, READ E_01 and REPEAT THE QUESTION	Ir a Q_03_4<br><br>OTRO, LEE E_01 y REPETIR LA PREGUNTA.
Q_03_4	Demo	What is your highest education level? If you don't have any studies press 1, if your highest educational level is elementary school press 2, if it is high school press 3, if it is a technical degree press 4, if you have an undergraduate or graduate degree press 5	¿Cuál es el nivel educativo más alto alcanzado por usted?<br>Si usted no ha cursado ningún grado presione 1;<br>Si cursó algún grado de primaria presione 2;<br>Si cursó algún grado de secundaria o bachillerato presione 3;<br>Si cursó un grado técnico o tecnológico presione 4;<br>Si usted estudió un grado universitario presione 5.	1= I don't have any studies<br><br>2= My highest educational level is elementary school<br><br>3= My highest educational level is middle or high school<br><br>4= I have a technical or technological degree<br><br>5= I have an undergraduate or graduate degree	1 = Ninguno<br>2 = Primaria<br>3 = Secundaria<br>4 = Técnico o Tecnológico<br>5 = Universitario o posgrado	GO TO Q_04<br><br>OTHER, READ E_01 and REPEAT THE QUESTION	Ir a Q_04<br><br>OTRO, LEE E_01 y REPETIR LA PREGUNTA.
ELIGIBILITY FOR MESSAGING	NO AUDIO	NO AUDIO	NO AUDIO	NO AUDIO	NO AUDIO	CONTINUE WITH MESSAGE:<br><br>If Q_2_1<2<br><br>If Q_2_1=2 AND Q_2_2=5 AND Age>50	CONTINUAR CON MENSAJE:<br><br>Si Q_2_1<2<br><br>Si Q_2_1=2 Y Q_2_2=5 Y Age>50

						If Q_2_1=3 AND Q_2_2!=5 AND Age>50<br><br>GO TO Q_13<br><br>If Q_2_1=2 AND Q_2_2!=5 AND Age<50<br><br>If Q_2_1=3 AND Q_2_2!=5 AND Age<50<br><br>If Q_2_1=3 AND Q_2_2=5 AND Age>50<br><br>If Q_2_1 = 4	Si Q_2_1=3 Y Q_2_2!=5 Y Age>50<br><br>IR A Q_13<br><br>Si Q_2_1=2 Y Q_2_2!=5 Y Age<50<br><br>SI Q_2_1=3 Y Q_2_2!=5 Y Age<50<br><br>Si Q_2_1=3 Y Q_2_2=5 Y Age>50<br><br>Si Q_2_1 = 4
Q_04	Intro Message	Your responses have been very helpful and you have qualified to receive a brief series of messages on COVID-19 vaccination for the next two months. Every time we call you, we will send you a message that will take less than 5 minutes and will credit 4,000 pesos to your mobile phone account if you have a prepaid mobile phone.<br><br>If you accept, we will start today and will be calling you again later this week for a few weeks.<br><br>Do you like to participate? If YES, press 1. If NO, press 3	Sus respuestas han sido muy valiosas y gracias a estas le hemos elegido para participar en un corto programa nacional de mensajes sobre la vacunación contra COVID-19 los próximos dos meses. Cada vez que le llamemos le haremos una recarga de 4,000 pesos en su teléfono celular si es prepago.<br><br>Si usted acepta, empezaremos hoy con el primer mensaje y le haremos solo dos llamadas por semana.<br><br>Si le gustaría participar de este programa, presione 1,<br>Si no desea participar, por favor presione 3.	1 = YES<br>3 = NO<br>OTHER=E_01	1 = SÍ<br>3 = NO<br>OTRO = E_01	IF 1, GO TO Message<br><br>IF 3, GO TO Q_13	Si SI, presione 1<br><br>SI NO, presione 3
Message						GO TO Test	Ir a Test

Test						GO TO Q_13	Ir a Q_13
Q_13	Exit	Is your phone number prepaid or postpaid?<br>For prepaid, press 1.<br>For postpaid, press 3.	¿Su número telefónico es prepago o postpago?<br>Para prepago, presione 1.<br>Para postpago, presione 3.	1 = PREPAID<br>3 = POSTPAID<br>OTHER=E_01	1 = PREPAGO<br>3 = POSTPAGO<br>OTRO = E_01	IF 1, GO TO M_01<br><br>IF 3, GO TO M_03<br><br>OTHER, READ E_01 and REPEAT THE QUESTION	Si 1, ir a M_01.<br><br>Si 3, ir a M_03<br><br>OTRO, LEER E_01 y REPETIR LA PREGUNTA.
M_01	Thank You	You have given us very valuable information and we are sincerely grateful. We will credit your account if it is prepaid with 4,000 pesos, and will call you again soon	Usted nos ha provisto una información muy valiosa y estamos muy agradecidos. Le recargaremos su teléfono móvil, si es prepago, con 4,000 pesos y lo llamaremos de nuevo pronto.				
M_03	Thank You	You have given us very valuable information and we are sincerely grateful. We will call you again soon	Usted nos ha provisto una información muy valiosa y estamos muy agradecidos. Le llamaremos de nuevo pronto.				
M_04	Reconnect message	Hi, this is the Pontificia Universidad Javeriana de Bogota, and Johns Hopkins University calling you back. It seems that the call dropped. We'll continue the COVID-19 survey where you left off.<br><br>If you would like to continue with the survey, press 1. If you do not want to continue with the call, press 3.	Hola. La Pontificia Universidad Javeriana de Bogotá y la Universidad de Johns Hopkins le esta regresado la llamada para continuar con la encuesta sobre COVID-19 que usted estaba realizando. Al parecer la llamada se cayó.<br><br>Si desea continuar con la encuesta, presione 1. Si no desea continuar con la llamada, presione 3.	1: Continue with the survey.<br><br>3: Do not continue with the survey.	1: Continuar con la encuesta.<br><br>3: No continuar con la encuesta.	IF 1, CONTINUE THE SURVEY WHERE THEY LEFT OFF.<br><br>IF 3, GO TO M_01	SI 1, CONTINUAR LA ENCUESTA DONDE LA DEJARON.<br><br>Si 3, ir a M_01

Subsequent calls

Intro Message _2		This is the Universidad Javeriana and Johns Hopkins survey about COVID-19 vaccination. When finished the call, which will take less than 5 minutes, we will credit your prepaid mobile phone with 4,000 pesos. We will deliver a message and then will make a quick question about the message.<br><br>Do you accept to continue?	Hola. Esta es de nuevo la encuesta de la Universidad Javeriana y Johns Hopkins sobre vacunación para COVID-19.<br><br>Al finalizar la llamada, que tomará menos de cinco minutos, le haremos una recarga de 4,000 pesos en su teléfono celular prepago. Usted escuchará un breve mensaje sobre vacunación para	1 = YES<br>3 = NO<br>OTHER=E_01	1 = SÍ<br>3 = NO<br>OTRO = E_01	IF 1, GO TO VACCINE<br><br>IF 3, GO TO M_02	Si 1, Ir a Vaccine<br><br>SI 3, ir a M_02

		If YES, press 1. If NO, press 3	COVID-19 y luego le haremos una pregunta sobre el mismo.<br><br>¿Acepta continuar?<br><br>Si acepta, presione 1,<br>Si no acepta presione 3.				
Vaccine		Have you been vaccinated against COVID-19 in the last week? If YES, press 1. If NO, press 3	Se ha usted vacunado contra COVID-19 en la última semana? Si se ha vacunado contra COVID-19 en la última semana, presione 1. Si no se ha vacunado en la última semana, presione 3.	1 = YES<br>3 = NO<br>OTHER=E_01	1 = SÍ<br>3 = NO<br>OTRO = E_01	IF 1, GO TO M_02<br><br>IF 3, GO TO Message	Si 1, Ir a M_02<br><br>SI 3, Ir a Message
Message						GO TO Test	Ir a Test
Test						GO TO Pre_postpaid	ir a Pre_postpaid .
Pre_post paid	Pre or postpaid	Finally, we would like to confirm if your phone line is prepaid or postpaid.<br><br>For prepaid dial 1.<br>For postpaid dial 3.	Para finalizar, nos gustaría confirmar si su línea de teléfono es prepago o pospago.<br><br>Para prepago marque 1.<br>Para postpago marque 3.	1 = prepaid<br>3 = postpaid<br>OTHER=E_01	1 = prepaid<br>3 = postpaid<br>OTRO = E_01	IF 1, GO TO M_01<br><br>IF 3, GO TO M_03	Si 1, Ir a M_01<br>SI 3, Ir a M_03
M_01	Thank You	You have given us very valuable information and we are sincerely grateful. We will credit your account if it is prepaid with 4,000 pesos, and will call you again soon	Usted nos ha provisto una información muy valiosa y estamos muy agradecidos. Le recargaremos su teléfono móvil, si es prepago, con 4,000 pesos y lo llamaremos de nuevo pronto.				
M_02	Thank You	The survey has ended. Universidad Javeriana and Johns Hopkins thank you for responding the call. Remember to get the vaccine, wash your hands and use masks.	¡Muchas gracias, hemos terminado la encuesta! Las Universidades Javeriana y de Johns Hopkins le agradecemos por atender esta llamada. Recuerde vacunarse, lavarse las manos cada vez que pueda y usar su tapabocas.				

M_03	Thank You	You have given us very valuable information and we are sincerely grateful. We will call you again soon	Usted nos ha provisto una información muy valiosa y estamos muy agradecidos. Le llamaremos de nuevo pronto.				
M_04	Reconnect message	Hi, this is the Pontificia Universidad Javeriana de Bogota, and Johns Hopkins University calling you back. It seems that the call dropped. We'll continue the COVID-19 survey where you left off.<br><br>If you would like to continue with the survey, press 1. If you do not want to continue with the call, press 3.	Hola. La Pontificia Universidad Javeriana de Bogotá y la Universidad de Johns Hopkins le esta regresado la llamada para continuar con la encuesta sobre COVID-19 que usted estaba realizando. Al parecer la llamada se cayó.<br><br>Si desea continuar con la encuesta, presione 1. Si no desea continuar con la llamada, presione 3.	1: Continue with the survey.<br><br>3: Do not continue with the survey.	1: Continuar con la encuesta.<br><br>3: No continuar con la encuesta.	IF 1, CONTINUE THE SURVEY WHERE THEY LEFT OFF.<br><br>IF 3, GO TO M_01	SI 1, CONTINUAR LA ENCUESTA DONDE LA DEJARON.<br><br>Si 3, ir a M_01

	Spanish version	English version
**Semana/ week**	**Brazo 1: Mensajes fácticos**	**Arm 1: Factual messages**
	**Mensajes**	**Messages**
1	**Vacunación e infección**	**Vaccination and infection**
	¿Sabía usted que vacunarse contra COVID-19 reduce el riesgo de desarrollar síntomas graves, de necesitar hospitalización y de morir?<br><br>Las vacunas contra COVID-19, al igual que para otras enfermedades, ayudan a que, si te enfermas, los síntomas sean más suaves que si no tuvieras la vacuna.<br><br>Por ejemplo, de acuerdo a datos del Ministerio de Salud de Colombia, las personas que han sido vacunadas contra COVID-19, tienen tres veces menos riesgo de hospitalización que las personas no vacunadas.<br><br>Si aún no se ha vacunado o le falta alguna dosis, recuerde hacerlo lo antes posible.	Did you know that getting vaccinated against COVID-19 reduces the risk of developing severe symptoms, needing hospitalization, and dying?<br><br>Vaccinations against COVID-19, as for other diseases, help make it so that if you get sick, your symptoms are milder than if you did not have the vaccine.<br><br>For example, according to data from the Colombian Ministry of Health, people who have been vaccinated against COVID-19 have three times less risk of hospitalization than unvaccinated people.<br><br>If you have not yet been vaccinated or are missing a dose, remember to do so as soon as possible.
2	**Efectividad de las vacunas**	**Effectiveness of vaccines**
	¿Sabía usted que a junio de 2022, 8 de cada 10 colombianos se había aplicado al menos una dosis de la vacuna contra COVID-19 y que, gracias a esta, se han salvado más de 36,000 vidas?<br><br>Además, más de la mitad de las personas vacunadas contra COVID-19 no necesitaron hospitalización cuando se contagiaron.<br><br>Si aún no se ha vacunado, recuerde hacerlo pronto porque estamos en un nuevo pico de COVI-19. Si le acaba de dar COVID-19, recuerde esperar 30 días para vacunarse.<br><br>Es necesario vacunarse porque las defensas que generó su cuerpo durante esa infección, no serán suficientes para combatir un nuevo ataque del virus.	Did you know that by June 2022, 8 out of 10 Colombians had received at least one dose of the COVID-19 vaccine and that more than 36,000 lives have been saved as a result?<br><br>In addition, more than half of the people vaccinated against COVID-19 did not need hospitalization when they were infected.<br><br>If you have not yet been vaccinated, remember to do so soon because we are in a new COVI-19 peak. If you have just been given COVID-19, remember to wait 30 days to get vaccinated.<br><br>It is necessary to get vaccinated because the defenses your body generated during that infection will not be enough to fight off a new attack by the virus.
3	**Proceso de desarrollo de las vacunas**	**Vaccine development process**

	¿Sabía usted que las vacunas contra COVID-19 se desarrollaron siguiendo rigurosos procesos de aprobación y monitoreo de su seguridad en todo el mundo?<br><br>Fue más rápido crear la vacuna contra COVID-19 que para otras enfermedades porque tenemos más tecnología, porque ya había estudios científicos para diseñar vacunas contra otras enfermedades parecidas y, porque esta vez la comunidad científica de todo el mundo trabajó colaborativamente. Gracias a todo esto, a junio de 2022 más de 42 millones de personas en Colombia han sido vacunadas con al menos una dosis de la vacuna contra COVID-19.<br><br>Vacunándonos nos protegemos todos. Si aún no se ha vacunado o le falta alguna dosis, recuerde hacerlo lo antes posible.	Did you know that the COVID-19 vaccines were developed following rigorous approval and safety monitoring processes around the world?<br><br>It was faster to create the vaccine against COVID-19 than for other diseases because we have more technology, because there were already scientific studies to design vaccines against other similar diseases, and because this time the scientific community around the world worked collaboratively. Thanks to all this, by June 2022 more than 42 million people in Colombia have been vaccinated with at least one dose of the vaccine against COVID-19.<br><br>By getting vaccinated, we all protect ourselves. If you have not yet been vaccinated or are missing a dose, remember to do it as soon as possible.
4	**Ingredientes**	**Ingredients**
	¿Alguna vez se ha preguntado de qué están hechas las vacunas contra COVID-19?<br><br>Sin importar la marca, porque todas sirven y son seguras, éstas vacunas no contienen huevos, gluten, ni preservativos. Tampoco tienen metales, plásticos, ni equipos electrónicos. Las vacunas le enseñan al cuerpo a generar defensas contra futuros ataques del virus.<br><br>Si aún no se ha vacunado contra COVID-19 o le falta alguna dosis, recuerde hacerlo lo antes posible	Have you ever wondered what COVID-19 vaccines are made?<br><br>Regardless of the brand, because they are all good and safe, these vaccines do not contain eggs, gluten, or preservatives. They are also free of metals, plastics, and electronic equipment. The vaccines teach the body to build defenses against future attacks by the virus.<br><br>If you have not yet been vaccinated against COVID-19 or are missing a dose, remember to do it as soon as possible.
5	**Seguridad y efectos adversos**	**Safety and adverse effects**
	¿Sabía usted que las vacunas contra COVID-19 han evitado que tres de cada cinco personas infectadas con COVID-19 sean hospitalizadas?<br><br>Pero, vacunarse, como cualquier cambio que se hace en el cuerpo puede tener un efecto.<br><br>En los estudios que se hicieron para garantizar	Did you know that COVID-19 vaccines have prevented three out of five people infected with COVID-19 from being hospitalized?<br><br>But, getting vaccinated, like any change you make to your body, can have an effect.

	que las vacunas fueran seguras, donde participaron miles de personas, se encontró que algunas personas reportaron efectos temporales de las vacunas entre estos, dolor transitorio en el brazo en el que se aplicó la vacuna, un poco de cansancio, fiebre o náuseas y en algunas mujeres, alteraciones en la menstruación.<br><br>Recuerde que los posibles malestares causados por la vacuna son temporales y que los beneficios de la vacuna son aún mayores. Podría salvarle la vida.<br><br>Si aún no se ha vacunado o le falta alguna dosis, recuerde hacerlo lo antes posible.	In studies done to ensure that the vaccines were safe, involving thousands of people, it was found that some people reported temporary effects of the vaccines, including temporary pain in the arm where the vaccine was given, some tiredness, fever or nausea, and in some women, alterations in menstruation.<br><br>Remember that the possible discomfort caused by the vaccine is temporary and that the benefits of the vaccine are even greater. It could save your life.<br><br>If you have not yet been vaccinated or are missing a dose, remember to do it as soon as possible.
6	**Dosis de refuerzo**	**Booster dose**
	¿Sabía usted que las vacunas de refuerzo contra COVID-19, ayudan a mantener la protección contra los sintomas graves de la enfermedad?<br><br>Cuando nos aplicamos las dosis de refuerzo, incrementamos la inmunidad de nuestro cuerpo y su capacidad para combatir un nuevo ataque del virus. Esto es importante porque así como sucede con otras vacunas, la protección de las vacunas contra COVID-19 va disminuyendo en la medida que pasa el tiempo y la enfermedad va cambiando.<br><br>Recuerde que la primera dosis de refuerzo está disponible para todas las personas mayores de 18 años y, se aplica cuatro meses después de haber completado el esquema de vacunación.<br><br>Si le falta la dosis de refuerzo, recuerde hacerlo lo antes posible.	Did you know that COVID-19 booster vaccines help maintain protection against severe symptoms of the disease?<br><br>When we get booster doses, we increase our body's immunity and its ability to fight off a new attack by the virus. This is important because as with other vaccines, the protection of the COVID-19 vaccines decreases as time passes and the disease changes.<br><br>Remember that the first booster dose is available to everyone 18 years of age and older and is given four months after the vaccination schedule is completed.<br><br>If you are missing the booster dose, remember to get it as soon as possible.
7	**Protección y cuidado a la familia**	**Family protection and care**
	¿Sabía usted que estudios realizados en Colombia y otros países han encontrado que las vacunas contra COVID-19 ayudan a proteger a los adultos, adolescentes, niños y niñas con quienes convivimos?	Did you know that studies conducted in Colombia and other countries have found that vaccines against COVID-19 help protect adults, adolescents, boys and girls with whom we live?

	Gracias a estas vacunas, el año pasado se salvaron más de 36,000 vidas en Colombia.<br><br>Vacunándonos nos protegemos todos. Si aún no se ha vacunado o le falta alguna dosis, recuerde hacerlo lo antes posible.	Thanks to these vaccines, more than 36,000 lives were saved last year in Colombia.<br><br>By getting vaccinated, we all protect ourselves. If you have not yet been vaccinated or are missing a dose, remember to do it as soon as possible.

	Spanish version	English version
**Semana/ week**	**Brazo 2: Mensajes narrativos**	**Arm 1: Narrative messages**
	**Mensajes**	**Messages**
1	**Vacunación e infección**	**Vaccination and infection**
	Oír para creer, entrevistamos a personas de todo Colombia y, ¿saben qué nos contaron sobre la vacunación contra COVID-19?<br><br>"Al principio de la pandemia fue un poco difícil, uno venía acostumbrado a una rutina de trabajo, vida social y, pues, de repente, por cuestiones de salud, todo cambió. Pero, precisamente, la vacuna es para que, si llegado el caso te llegas a contagiar, no te pegue tan fuerte".<br><br>Las vacunas reducen el riesgo de desarrollar síntomas graves. Si aún no te has vacunado o te falta alguna dosis, recuerda visitar el punto de vacunación más cercano.	Hearing to Believe, we interviewed people from all over Colombia and, do you know what they told us about the vaccination against COVID-19?<br><br>"At the beginning of the pandemic it was a little difficult, one was used to a routine of work, social life and, well, suddenly, due to health issues, everything changed. But, precisely, the vaccine is so that, if you do get infected, it won't hit you so hard".<br><br>Vaccinations reduce the risk of developing serious symptoms. If you have not yet been vaccinated or if you are missing a dose, remember to visit the nearest vaccination center.
2	**Efectividad de las vacunas y vacunación e infección**	**Effectiveness of vaccines and vaccination and infection.**
	Oír para creer, entrevistamos a personas de todo Colombia y, ¿saben qué nos contaron sobre la vacunación contra COVID-19?<br><br>“A mí me dio dos veces COVID y me dejó secuelas: con muchos problemas respiratorios y ahora estoy en tratamiento. Entonces, para poder vacunarme tuve que preguntarle a mis médicos y ellos me dijeron que no había ningún problema, así que me vacuné con dos dosis y estoy pendiente de la tercera. Y le cuento que, para mi sorpresa, pensé que por mis secuelas iba a tener alguna reacción por la vacuna y ¡oiga, no! no me dio nada."<br><br>Así te hayas enfermado de COVID-19 también puedes vacunarte. Si aún no te has vacunado o te falta alguna dosis, recuerda visitar el punto de vacunación más cercano.	Hearing to Believe, we interviewed people from all over Colombia and, do you know what they told us about the vaccination against COVID-19?<br><br>“I got COVID twice, and it left me with sequelae: many respiratory problems, and now I am in treatment. So, in order to get vaccinated, I had to ask my doctors, and they told me that there was no problem, so I got vaccinated with two doses, and I am waiting for the third one. And I tell you, to my surprise, I thought that because of my sequelae, I was going to have some reaction from the vaccine, and hey, no! I didn't get anything."<br><br>Even if you have been sick with COVID-19, you can also get vaccinated. If you have not yet been vaccinated or are missing a dose, remember to visit the nearest vaccination point.
3	**Proceso de desarrollo de las vacunas**	**Vaccine development process**

	Oír para creer, entrevistamos a personas de todo Colombia y, ¿saben qué nos contaron sobre la vacunación contra COVID-19?<br><br>“La vacunación, para mí, es algo muy importante. Realmente es que con la crisis que hubo, ¡fue muy impresionante que hayan sacado tan rápido las vacunas! Cuando me enteré de las vacunas, me sentí feliz, porque, afortunadamente, a pesar de estar trabajando en un hospital, no me contagié. Ya con la vacuna estaba más tranquila en mi trabajo. Soy instrumentadora quirúrgica, y siempre he creído en la ciencia, porque ella está disponible para nosotros, para actuar rápido ante estas situaciones."<br><br>Las vacunas funcionan. Si aún no te has vacunado o te falta alguna dosis, recuerda visitar el punto de vacunación más cercano.	Hearing to Believe, we interviewed people from all over Colombia and, do you know what they told us about the vaccination against COVID-19?<br><br>"Vaccination, for me, is something very important. Really, with the crisis that there was, it was very impressive that they brought out the vaccines so quickly! When I found out about the vaccines, I was happy because, fortunately, despite working in a hospital, I didn't get infected. With the vaccine, I was already calmer at work. I am a surgical instrument maker, and I have always believed in science because it is available to us to act quickly in these situations."<br><br>Vaccines work. If you have not yet been vaccinated or if you are missing a dose, remember to visit the nearest vaccination point.
4	**Protección de la sociedad y responsabilidad social**	**Protection of society and social responsibility**
	Oír para creer, entrevistamos a personas de todo Colombia y, ¿saben qué nos contaron sobre la vacunación contra COVID-19?<br><br>“Mi decisión siempre fue vacunarme por prevención, por salud, por respeto a mi familia y a nivel social, porque creo que es algo de responsabilidad social, que todos debemos estar vacunados; considero que todos tenemos un riesgo, no sabemos cómo va a reaccionar el virus en cada persona y bueno, creo que debería motivarnos, principalmente, el amor a nuestra familia y a nosotros mismos, y por eso, en mi caso, debo estar bien y protegerlos de cierta manera; entonces, ya tengo las tres dosis de la vacuna y súper bien, no tuve ningún tipo de reacción en ninguna de las tres dosis. También mi familia está vacunada y ninguno tuvo síntomas".<br><br>Cuidarnos es responsabilidad de todos. Si aún no te has vacunado o te falta alguna dosis,	Hearing to Believe, we interviewed people from all over Colombia and, do you know what they told us about the vaccination against COVID-19?<br><br>"My decision was always to get vaccinated for prevention, for health, out of respect for my family. And at a social level because it is something of social responsibility that we should all be vaccinated. I consider that we all have a risk, we do not know how the virus will react in each person, and well, I think we should be motivated, mainly, by love for our family and ourselves, and therefore, in my case, I must be well and protect them in a certain way; then, I already have the three doses of the vaccine, and super well, I did not have any kind of reaction in any of the three doses. My family is also vaccinated, and none of them had any symptoms".

	recuerda visitar el punto de vacunación más cercano.	Taking care of ourselves is everyone's responsibility. If you have not yet been vaccinated or if you are missing any dose, remember to visit the nearest vaccination point.
5	**Seguridad y efectos adversos**	**Safety and adverse effects**
	Oír para creer, entrevistamos a personas de todo Colombia y, ¿saben qué nos contaron sobre la vacunación contra COVID-19?<br><br>“En este momento estoy desempleada porque debido a la pandemia el colegio donde yo trabajaba tuvo que cerrar. La pandemia nos cambió la vida 100%... y la vacunación fue una manera de sobrevivir a este virus, porque sabíamos que si nos daba, podríamos sobrevivir con la vacuna, entonces, por el bienestar de todos, en mi familia todos estamos vacunados. En mi caso, tengo todas las dosis y la de refuerzo. Tuve miedo de ponerme esta última por todos los comentarios que escuchaba de esa vacuna, pero no, normal. Nos fue super bien a mi esposo y a mí que nos la pusimos."<br><br>Las vacunas son seguras. Si aún no te has vacunado o te falta alguna dosis, recuerda visitar el punto de vacunación más cercano.	Hearing to Believe, we interviewed people from all over Colombia and, do you know what they told us about the vaccination against COVID-19?<br><br>"At the moment I am unemployed because due to the pandemic the school where I worked had to close. The pandemic changed our lives 100%... and vaccination was a way to survive this virus because we knew that if it hit us, we could survive with the vaccine, so, for everyone's wellbeing, in my family, we are all vaccinated. In my case, I have all the doses and the booster. I was afraid to get the booster because of all the comments I heard about this vaccine, but no, normal. It went super well for my husband and me, who got it."<br><br>Vaccines are safe. If you have not yet been vaccinated or are missing a dose, remember to visit your nearest vaccination site.
6	**Libertad para elegir y seguridad**	**Freedom of choice and security**
	Oír para creer, entrevistamos a personas de todo Colombia y, ¿saben qué nos contaron sobre la vacunación contra COVID-19?<br><br>“La vacuna ha servido mucho, después de la vacunación, disminuyó el número de muertes por COVID-19. Entonces, creo yo que la gente debería vacunarse, pues, para estar más seguros, para vivir más tranquilos y seguir viviendo la cotidianidad; y para eso tenemos que poner de nuestra parte."<br><br>Vacunándonos contra COVID-19 estamos más seguros. Si aún no te has vacunado o te falta	Hearing to Believe, we interviewed people from all over Colombia and, do you know what they told us about the vaccination against COVID-19?<br><br>"The vaccine has been very useful. After the vaccination, the number of deaths due to COVID-19 decreased. So, people should get vaccinated to be safer, to live in peace, and continue living our daily lives; and for that we have to do our part.<br><br>By getting vaccinated against COVID-19 we are safer. If you have not yet been vaccinated or if you are missing a dose,

	alguna dosis, recuerda visitar el punto de vacunación más cercano	remember to visit the nearest vaccination point.
7	**Protección y cuidado a la familia**	**Family protection and care**
	Oír para creer, entrevistamos a personas de todo Colombia y, ¿saben qué nos contaron sobre la vacunación contra COVID-19?<br><br>“Mi hijo de cinco añitos ya está vacunado también, pues, porque tampoco quería que él se enfermara y como estaba yendo al colegio y salía con amiguitos, entonces, por eso. ¡Yo quiero tanto a mis hijos! Los adoro y daría la vida por ellos, y por eso me vacuné, porque que tal un día salga, me contagie, yo llegue y los abrace o los bese y les pase el virus… yo tenía que hacerlo por ellos."<br><br>Protégete y protege a tu familia. Si aún no te has vacunado o te falta alguna dosis, recuerda visitar el punto de vacunación más cercano.	Hearing to Believe, we interviewed people from all over Colombia and, do you know what they told us about the vaccination against COVID-19?<br><br>"My five-year-old son is already vaccinated too, because I did not want him to get sick either, and since he was going to school and going out with his friends, that's why I love my children so much! I adore them, and I would give my life for them, and that's why I got vaccinated because what if one day I go out, I get infected, I come and hug them or kiss them and pass the virus to them... I had to do it for them."<br><br>Protect yourself and your family. If you have not yet been vaccinated or are missing a dose, remember to visit the nearest vaccination point.

	Spanish version	English version	Spanish version	English version
**Semana/ week**	**Brazo 3: Mensajes mixtos**	**Arm 3: Mixed messages**	**Brazo 3: Mensajes mixtos**	**Arm 3: Mixed messages**
	**Mensajes día 1**	**Messages day 1**	**Mensajes día 2**	**Messages day 2**
1	**Vacunación e infección**	**Vaccination and infection**	**Vacunación e infección**	**Vaccination and infection**
	¿Sabía usted que vacunarse contra COVID-19 reduce el riesgo de desarrollar síntomas graves, de necesitar hospitalización y de morir?<br><br>Las vacunas contra COVID-19, al igual que para otras enfermedades, ayudan a que, si te enfermas, los síntomas sean más suaves que si no tuvieras la vacuna.<br><br>Por ejemplo, de acuerdo a datos del Ministerio de Salud de Colombia, las personas que han sido vacunadas contra COVID-19, tienen tres veces menos riesgo de hospitalización que las personas no vacunadas.<br><br>Si aún no se ha vacunado o le falta alguna dosis, recuerde hacerlo lo antes posible.	Did you know that getting vaccinated against COVID-19 reduces the risk of developing severe symptoms, needing hospitalization, and dying?<br><br>Vaccinations against COVID-19, as for other diseases, help make it so that if you get sick, your symptoms are milder than if you did not have the vaccine.<br><br>For example, according to data from the Colombian Ministry of Health, people who have been vaccinated against COVID-19 have three times less risk of hospitalization than unvaccinated people.<br><br>If you have not yet been vaccinated or are missing a dose, remember to do so as soon as possible.	Oír para creer, entrevistamos a personas de todo Colombia y, ¿saben qué nos contaron sobre la vacunación contra COVID-19?<br><br>"Al principio de la pandemia fue un poco difícil, uno venía acostumbrado a una rutina de trabajo, vida social y, pues, de repente, por cuestiones de salud, todo cambió. Pero, precisamente, la vacuna es para que, si llegado el caso te llegas a contagiar, no te pegue tan fuerte".<br><br>Las vacunas reducen el riesgo de desarrollar síntomas graves. Si aún no te has vacunado o te falta alguna dosis, recuerda visitar el punto de vacunación más cercano.	Hearing to Believe, we interviewed people from all over Colombia and, do you know what they told us about the vaccination against COVID-19?<br><br>"At the beginning of the pandemic it was a little difficult, one was used to a routine of work, social life and, well, suddenly, due to health issues, everything changed. But, precisely, the vaccine is so that, if you do get infected, it won't hit you so hard".<br><br>Vaccinations reduce the risk of developing serious symptoms. If you have not yet been vaccinated or if you are missing a dose, remember to visit the nearest vaccination center.

2	Efectividad de las vacunas	Effectiveness of vaccines	Efectividad de las vacunas y vacunación e infección	Effectiveness of vaccines and vaccination and infection.
	¿Sabía usted que a junio de 2022, 8 de cada 10 colombianos se había aplicado al menos una dosis de la vacuna contra COVID-19 y que, gracias a esta, se han salvado más de 36,000 vidas?<br><br>Además, más de la mitad de las personas vacunadas contra COVID-19 no necesitaron hospitalización cuando se contagiaron.<br><br>Si aún no se ha vacunado, recuerde hacerlo pronto porque estamos en un nuevo pico de COVI-19. Si le acaba de dar COVID-19, recuerde esperar 30 días para vacunarse.<br><br>Es necesario vacunarse porque las defensas que generó su cuerpo durante esa infección, no serán suficientes para combatir un nuevo ataque del virus.	Did you know that by June 2022, 8 out of 10 Colombians had received at least one dose of the COVID-19 vaccine and that more than 36,000 lives have been saved as a result?<br><br>In addition, more than half of the people vaccinated against COVID-19 did not need hospitalization when they were infected.<br><br>If you have not yet been vaccinated, remember to do so soon because we are in a new COVI-19 peak. If you have just been given COVID-19, remember to wait 30 days to get vaccinated.<br><br>It is necessary to get vaccinated because the defenses your body generated during that infection will not be enough to fight off a new attack by the virus.	Oír para creer, entrevistamos a personas de todo Colombia y, ¿saben qué nos contaron sobre la vacunación contra COVID-19?<br><br>“A mí me dio dos veces COVID y me dejó secuelas: con muchos problemas respiratorios y ahora estoy en tratamiento. Entonces, para poder vacunarme tuve que preguntarle a mis médicos y ellos me dijeron que no había ningún problema, así que me vacuné con dos dosis y estoy pendiente de la tercera. Y le cuento que, para mi sorpresa, pensé que por mis secuelas iba a tener alguna reacción por la vacuna y ¡oiga, no! no me dio nada."<br><br>Así te hayas enfermado de COVID-19 también puedes vacunarte. Si aún no te has vacunado o te falta alguna dosis, recuerda visitar el punto de vacunación más cercano.	Hearing to Believe, we interviewed people from all over Colombia and, do you know what they told us about the vaccination against COVID-19?<br><br>“I got COVID twice, and it left me with sequelae: many respiratory problems, and now I am in treatment. So, in order to get vaccinated, I had to ask my doctors, and they told me that there was no problem, so I got vaccinated with two doses, and I am waiting for the third one. And I tell you, to my surprise, I thought that because of my sequelae, I was going to have some reaction from the vaccine, and hey, no! I didn't get anything."<br><br>Even if you have been sick with COVID-19, you can also get vaccinated. If you have not yet been vaccinated or are missing a dose, remember to visit the nearest vaccination point.

3	**Proceso de desarrollo de las vacunas**	**Vaccine development process**	**Proceso de desarrollo de las vacunas**	**Vaccine development process**
	¿Sabía usted que las vacunas contra COVID-19 se desarrollaron siguiendo rigurosos procesos de aprobación y monitoreo de su seguridad en todo el mundo?<br><br>Fue más rápido crear la vacuna contra COVID-19 que para otras enfermedades porque tenemos más tecnología, porque ya había estudios científicos para diseñar vacunas contra otras enfermedades parecidas y, porque esta vez la comunidad científica de todo el mundo trabajó colaborativamente. Gracias a todo esto, a junio de 2022 más de 42 millones de personas en Colombia han sido vacunadas con al menos una dosis de la vacuna contra COVID-19.<br><br>Vacunándonos nos protegemos todos. Si aún no se ha vacunado o le falta alguna dosis, recuerde hacerlo lo antes posible.	Did you know that the COVID-19 vaccines were developed following rigorous approval and safety monitoring processes around the world?<br><br>It was faster to create the vaccine against COVID-19 than for other diseases because we have more technology, because there were already scientific studies to design vaccines against other similar diseases, and because this time the scientific community around the world worked collaboratively. Thanks to all this, by June 2022 more than 42 million people in Colombia have been vaccinated with at least one dose of the vaccine against COVID-19.<br><br>By getting vaccinated, we all protect ourselves. If you have not yet been vaccinated or are missing a dose, remember to do it as soon as possible.	Oír para creer, entrevistamos a personas de todo Colombia y, ¿saben qué nos contaron sobre la vacunación contra COVID-19?<br><br>"La vacunación, para mí, es algo muy importante. Realmente es que con la crisis que hubo, ¡fue muy impresionante que hayan sacado tan rápido las vacunas! Cuando me enteré de las vacunas, me sentí feliz, porque, afortunadamente, a pesar de estar trabajando en un hospital, no me contagié. Ya con la vacuna estaba más tranquila en mi trabajo. Soy instrumentadora quirúrgica, y siempre he creído en la ciencia, porque ella está disponible para nosotros, para actuar rápido ante estas situaciones."<br><br>Las vacunas funcionan. Si aún no te has vacunado o te falta alguna dosis, recuerda visitar el punto de vacunación más cercano.	Hearing to Believe, we interviewed people from all over Colombia and, do you know what they told us about the vaccination against COVID-19?<br><br>"Vaccination, for me, is something very important. Really, with the crisis that there was, it was very impressive that they brought out the vaccines so quickly! When I found out about the vaccines, I was happy because, fortunately, despite working in a hospital, I didn't get infected. With the vaccine, I was already calmer at work. I am a surgical instrument maker, and I have always believed in science because it is available to us to act quickly in these situations."<br><br>Vaccines work. If you have not yet been vaccinated or if you are missing a dose, remember to visit the nearest vaccination point.

4	**Ingredientes**	**Ingredients**	**Protección de la sociedad y responsabilidad social**	**Protection of society and social responsibility**
	¿Alguna vez se ha preguntado de qué están hechas las vacunas contra COVID-19?<br><br>Sin importar la marca, porque todas sirven y son seguras, éstas vacunas no contienen huevos, gluten, ni preservativos. Tampoco tienen metales, plásticos, ni equipos electrónicos. Las vacunas le enseñan al cuerpo a generar defensas contra futuros ataques del virus.<br><br>Si aún no se ha vacunado contra COVID-19 o le falta alguna dosis, recuerde hacerlo lo antes posible	Have you ever wondered what COVID-19 vaccines are made?<br><br>Regardless of the brand, because they are all good and safe, these vaccines do not contain eggs, gluten, or preservatives. They are also free of metals, plastics, and electronic equipment. The vaccines teach the body to build defenses against future attacks by the virus.<br><br>If you have not yet been vaccinated against COVID-19 or are missing a dose, remember to do it as soon as possible.	Oír para creer, entrevistamos a personas de todo Colombia y, ¿saben qué nos contaron sobre la vacunación contra COVID-19?<br><br>“Mi decisión siempre fue vacunarme por prevención, por salud, por respeto a mi familia y a nivel social, porque creo que es algo de responsabilidad social, que todos debemos estar vacunados; considero que todos tenemos un riesgo, no sabemos cómo va a reaccionar el virus en cada persona y bueno, creo que debería motivarnos, principalmente, el amor a nuestra familia y a nosotros mismos, y por eso, en mi caso, debo estar bien y protegerlos de cierta manera; entonces, ya tengo las tres dosis de la vacuna y súper bien, no tuve ningún tipo de reacción en ninguna de las tres dosis. También mi familia está vacunada y ninguno tuvo síntomas".<br><br>Cuidarnos es responsabilidad de todos. Si aún no te has vacunado o te falta alguna dosis, recuerda visitar el punto de vacunación más cercano.	Hearing to Believe, we interviewed people from all over Colombia and, do you know what they told us about the vaccination against COVID-19?<br><br>"My decision was always to get vaccinated for prevention, for health, out of respect for my family. And at a social level because it is something of social responsibility that we should all be vaccinated. I consider that we all have a risk, we do not know how the virus will react in each person, and well, I think we should be motivated, mainly, by love for our family and ourselves, and therefore, in my case, I must be well and protect them in a certain way; then, I already have the three doses of the vaccine, and super well, I did not have any kind of reaction in any of the three doses. My family is also vaccinated, and none of them had any symptoms".<br><br>Taking care of ourselves is everyone's responsibility. If you have not yet been vaccinated or if you are missing any dose, remember to visit the nearest vaccination point.

5	**Seguridad y efectos adversos**	**Safety and adverse effects**	**Seguridad y efectos adversos**	**Safety and adverse effects**
	¿Sabía usted que las vacunas contra COVID-19 han evitado que tres de cada cinco personas infectadas con COVID-19 sean hospitalizadas?<br><br>Pero, vacunarse, como cualquier cambio que se hace en el cuerpo puede tener un efecto.<br><br>En los estudios que se hicieron para garantizar que las vacunas fueran seguras, donde participaron miles de personas, se encontró que algunas personas reportaron efectos temporales de las vacunas entre estos, dolor transitorio en el brazo en el que se aplicó la vacuna, un poco de cansancio, fiebre o náuseas y en algunas mujeres, alteraciones en la menstruación.<br><br>Recuerde que los posibles malestares causados por la vacuna son temporales y que los beneficios de la vacuna son aún mayores. Podría salvarle la vida.<br><br>Si aún no se ha vacunado o le falta alguna dosis, recuerde hacerlo lo antes posible.	Did you know that COVID-19 vaccines have prevented three out of five people infected with COVID-19 from being hospitalized?<br><br>But, getting vaccinated, like any change you make to your body, can have an effect.<br><br>In studies done to ensure that the vaccines were safe, involving thousands of people, it was found that some people reported temporary effects of the vaccines, including temporary pain in the arm where the vaccine was given, some tiredness, fever or nausea, and in some women, alterations in menstruation.<br><br>Remember that the possible discomfort caused by the vaccine is temporary and that the benefits of the vaccine are even greater. It could save your life.<br><br>If you have not yet been vaccinated or are missing a dose, remember to do it as soon as possible.	Oír para creer, entrevistamos a personas de todo Colombia y, ¿saben qué nos contaron sobre la vacunación contra COVID-19?<br><br>“En este momento estoy desempleada porque debido a la pandemia el colegio donde yo trabajaba tuvo que cerrar. La pandemia nos cambió la vida 100%... y la vacunación fue una manera de sobrevivir a este virus, porque sabíamos que si nos daba, podríamos sobrevivir con la vacuna, entonces, por el bienestar de todos, en mi familia todos estamos vacunados. En mi caso, tengo todas las dosis y la de refuerzo. Tuve miedo de ponerme esta última por todos los comentarios que escuchaba de esa vacuna, pero no, normal. Nos fue super bien a mi esposo y a mí que nos la pusimos."<br><br>Las vacunas son seguras. Si aún no te has vacunado o te falta alguna dosis, recuerda visitar el punto de vacunación más cercano.	Hearing to Believe, we interviewed people from all over Colombia and, do you know what they told us about the vaccination against COVID-19?<br><br>"At the moment I am unemployed because due to the pandemic the school where I worked had to close. The pandemic changed our lives 100%... and vaccination was a way to survive this virus because we knew that if it hit us, we could survive with the vaccine, so, for everyone's wellbeing, in my family, we are all vaccinated. In my case, I have all the doses and the booster. I was afraid to get the booster because of all the comments I heard about this vaccine, but no, normal. It went super well for my husband and me, who got it."<br><br>Vaccines are safe. If you have not yet been vaccinated or are missing a dose, remember to visit your nearest vaccination site.

6	**Dosis de refuerzo**	**Booster dose**	**Libertad para elegir y seguridad**	**Freedom of choice and security**
	¿Sabía usted que las vacunas de refuerzo contra COVID-19, ayudan a mantener la protección contra los sintomas graves de la enfermedad?<br><br>Cuando nos aplicamos las dosis de refuerzo, incrementamos la inmunidad de nuestro cuerpo y su capacidad para combatir un nuevo ataque del virus. Esto es importante porque así como sucede con otras vacunas, la protección de las vacunas contra COVID-19 va disminuyendo en la medida que pasa el tiempo y la enfermedad va cambiando.<br><br>Recuerde que la primera dosis de refuerzo está disponible para todas las personas mayores de 18 años y, se aplica cuatro meses después de haber completado el esquema de vacunación.<br><br>Si le falta la dosis de refuerzo, recuerde hacerlo lo antes posible.	Did you know that COVID-19 booster vaccines help maintain protection against severe symptoms of the disease?<br><br>When we get booster doses, we increase our body's immunity and its ability to fight off a new attack by the virus. This is important because as with other vaccines, the protection of the COVID-19 vaccines decreases as time passes and the disease changes.<br><br>Remember that the first booster dose is available to everyone 18 years of age and older and is given four months after the vaccination schedule is completed.<br><br>If you are missing the booster dose, remember to get it as soon as possible.	Oír para creer, entrevistamos a personas de todo Colombia y, ¿saben qué nos contaron sobre la vacunación contra COVID-19?<br><br>“La vacuna ha servido mucho, después de la vacunación, disminuyó el número de muertes por COVID-19. Entonces, creo yo que la gente debería vacunarse, pues, para estar más seguros, para vivir más tranquilos y seguir viviendo la cotidianidad; y para eso tenemos que poner de nuestra parte."<br><br>Vacunándonos contra COVID-19 estamos más seguros. Si aún no te has vacunado o te falta alguna dosis, recuerda visitar el punto de vacunación más cercano	Hearing to Believe, we interviewed people from all over Colombia and, do you know what they told us about the vaccination against COVID-19?<br><br>"The vaccine has been very useful. After the vaccination, the number of deaths due to COVID-19 decreased. So, people should get vaccinated to be safer, to live in peace, and continue living our daily lives; and for that we have to do our part.<br><br>By getting vaccinated against COVID-19 we are safer. If you have not yet been vaccinated or if you are missing a dose, remember to visit the nearest vaccination point.

7	**Protección y cuidado a la familia**	**Family protection and care**	**Protección y cuidado a la familia**	**Family protection and care**
	¿Sabía usted que estudios realizados en Colombia y otros países han encontrado que las vacunas contra COVID-19 ayudan a proteger a los adultos, adolescentes, niños y niñas con quienes convivimos?<br><br>Gracias a estas vacunas, el año pasado se salvaron más de 36,000 vidas en Colombia.<br><br>Vacunándonos nos protegemos todos. Si aún no se ha vacunado o le falta alguna dosis, recuerde hacerlo lo antes posible.	Did you know that studies conducted in Colombia and other countries have found that vaccines against COVID-19 help protect adults, adolescents, boys and girls with whom we live?<br><br>Thanks to these vaccines, more than 36,000 lives were saved last year in Colombia.<br><br>By getting vaccinated, we all protect ourselves. If you have not yet been vaccinated or are missing a dose, remember to do it as soon as possible.	Oír para creer, entrevistamos a personas de todo Colombia y, ¿saben qué nos contaron sobre la vacunación contra COVID-19?<br><br>“Mi hijo de cinco añitos ya está vacunado también, pues, porque tampoco quería que él se enfermara y como estaba yendo al colegio y salía con amiguitos, entonces, por eso. ¡Yo quiero tanto a mis hijos! Los adoro y daría la vida por ellos, y por eso me vacuné, porque que tal un día salga, me contagie, yo llegue y los abrace o los bese y les pase el virus… yo tenía que hacerlo por ellos."<br><br>Protégete y protege a tu familia. Si aún no te has vacunado o te falta alguna dosis, recuerda visitar el punto de vacunación más cercano.	Hearing to Believe, we interviewed people from all over Colombia and, do you know what they told us about the vaccination against COVID-19?<br><br>"My five-year-old son is already vaccinated too, because I did not want him to get sick either, and since he was going to school and going out with his friends, that's why I love my children so much! I adore them, and I would give my life for them, and that's why I got vaccinated because what if one day I go out, I get infected, I come and hug them or kiss them and pass the virus to them... I had to do it for them."<br><br>Protect yourself and your family. If you have not yet been vaccinated or are missing a dose, remember to visit the nearest vaccination point.
